# *Orthodenticle* Is Required for the Expression of Principal Recognition Molecules That Control Axon Targeting in the *Drosophila* Retina

**DOI:** 10.1371/journal.pgen.1005303

**Published:** 2015-06-26

**Authors:** Chiara Mencarelli, Franck Pichaud

**Affiliations:** MRC Laboratory for Molecular Cell Biology, University College London, London, United Kingdom; New York University, UNITED STATES

## Abstract

Parallel processing of neuronal inputs relies on assembling neural circuits into distinct synaptic-columns and layers. This is orchestrated by matching recognition molecules between afferent growth cones and target areas. Controlling the expression of these molecules during development is crucial but not well understood. The developing *Drosophila* visual system is a powerful genetic model for addressing this question. In this model system, the achromatic R1-6 photoreceptors project their axons in the lamina while the R7 and R8 photoreceptors, which are involved in colour detection, project their axons to two distinct synaptic-layers in the medulla. Here we show that the conserved homeodomain transcription factor Orthodenticle (Otd), which in the eye is a main regulator of *rhodopsin* expression, is also required for R1-6 photoreceptor synaptic-column specific innervation of the lamina. Our data indicate that *otd* function in these photoreceptors is largely mediated by the recognition molecules *flamingo* (*fmi*) and *golden goal* (*gogo*). In addition, we find that *otd* regulates synaptic-layer targeting of R8. We demonstrate that during this process, *otd* and the R8-specific transcription factor *senseless/Gfi1* (*sens*) function as independent transcriptional inputs that are required for the expression of *fmi*, *gogo* and the adhesion molecule *capricious* (*caps*), which govern R8 synaptic-layer targeting. Our work therefore demonstrates that *otd* is a main component of the gene regulatory network that regulates synaptic-column and layer targeting in the fly visual system.

## Author Summary

Establishing neural circuits during brain development relies on the ability for neurons to find their appropriate targets, a task that depends on recognition molecules whose expression must be tightly controlled. Very little is known of the factors that control the expression of these molecules. Here we have used the genetically amenable fly retina to address this issue. We identify Orthodenticle, a protein involved in mammalian brain development, as a main component of a new genetic cascade controlling the expression of recognition molecules that govern the wiring of the fly retina. Our work raises the issue that Orthodenticle might play a similar role in mammals. It also sheds new light on the genetic basis for wiring a model neural circuit during development.

## Introduction

**Fig 1:**
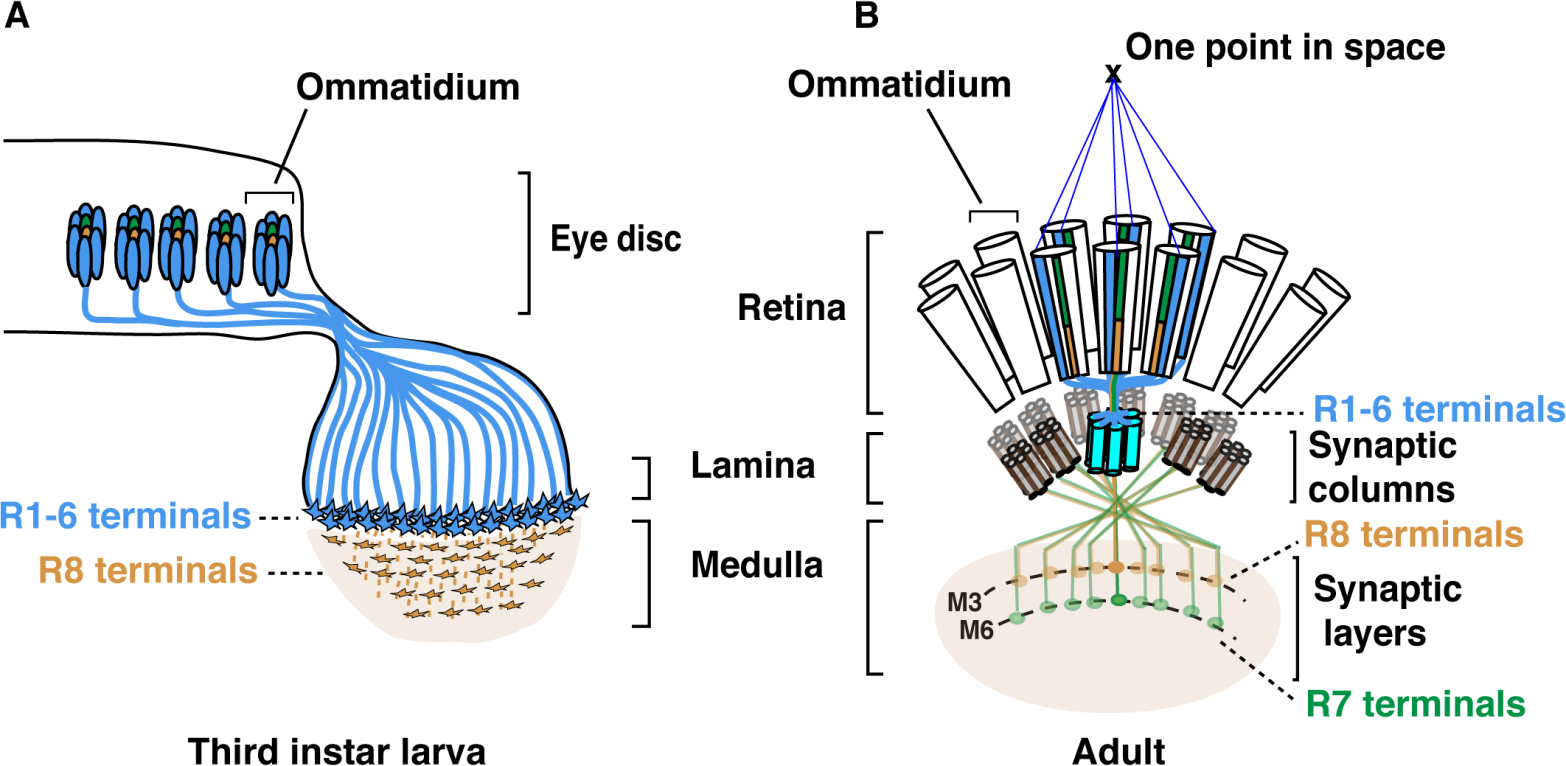
Schematic representation of the *Drosophila* visual system. **(A)** Schematic representation of *Drosophila* axonal photoreceptor projections in the third instar larva optic lobe. The outer R1-R6 (indicated in light blue) from each ommatidium in the eye disc project their axons into the lamina part of the brain. At this early developmental stage, the inner photoreceptor R8s (yellow) project through the lamina and establish a regular retinotopic array of terminals in the medulla. **(B)** Schematic representation of the adult *Drosophila* visual system. R-cell axons are organized into synaptic-columns and layers. Six different photoreceptors (indicated in light blue) from six neighbouring ommatidia share the same optical axis and pool their axons in the same synaptic-column in the lamina [2]. R8 (orange) and R7 (green) photoreceptor axons pass through the lamina and terminate in distinct synaptic-layers M3 (R8) and M6 (R7).

The *Drosophila* compound eye has long served as a powerful system to dissect the genetic and molecular basis for establishing neural circuits during development. Each of the 750 facet lenses (i.e., ommatidia) that form the fly eye contains six outer photoreceptors (R1-R6) and two inner photoreceptors (R7 and R8). R1-R6 are involved in motion detection and express the broad-spectrum Rhodopsin1 (Rh1) [1]. These photoreceptors project axons and form synapses in the lamina, a ganglion that lies directly below the retina. Neural wiring of the outer photoreceptors in the lamina follows the principle of neural superposition, where six neighbouring ommatidia each project one outer photoreceptor terminal to one given synaptic-column (Fig 1) [2,3]. R7 and R8 are involved in chromatic discrimination and express different *rhodopsin* genes. R7 photoreceptors express either the UV-rhodopsin *rh3* or *rh4* [4], whereas R8 photoreceptors express either the blue *rh5* or green-*rh6* [5,6]. Within each ommatidium, the light-gathering organelle of R7 shares its optical axis with that of the underlying R8 photoreceptor. Therefore, these two neurons can compare the chromatic composition of the incident light. In this context, each pair of R7/R8 axons is found within one column but their respective growth cones establish synaptic connections in distinct synaptic-layers (Fig 1B). R8 axons terminate within the superficial M3 layer and R7 axons terminate deeper in the medulla, in the M6 layer.

The layer-specific targeting of R7 and R8 photoreceptors is a dynamic process that follows a precise temporal sequence over the late third instar larval and pupal developmental stages (Fig 1 and S1 Fig). Within each ommatidium, R8 is the first photoreceptor to differentiate and to extend its axon toward the medulla. Approximately 24 h later, the R7 axons follow [7–9]. Later during pupal development, R8 and R7 growth cones are positioned in temporary layers, where they remain until the midpupal stage (S1 Fig). At this developmental stage, the growth cones of R7 and R8 regain motility and progress synchronously to their respective final synaptic-layers.

Several cell adhesion molecules (CAMs) and recognition molecules govern synaptic-column and-layer targeting of R1-6, R7 and R8 [10,11]. *fmi* [12], *gogo* [13], *NCadherin* (*NCad*) [14] and the *LAR* receptor tyrosine phosphatase [15] are required for R1-6 axons to select appropriate synaptic-columns in the lamina. Furthermore, *fmi* and *gogo* regulate proper spacing of the R8 axons and their targeting to the superficial medulla layer M1 [7]^,^[16]. Later in development, these two factors cooperate to direct the R8 terminals to their final destination, the M3 synaptic-layer [17]. In addition, the CAM leucine-rich-repeat molecule Caps also contributes to the layer targeting of R8, presumably by promoting adhesion between the R8 growth cones and medulla neurons in the recipient M3 layer [8]. In the case of R7, *NCad* [14] together with *LAR* [15] and the *Insulin Receptor* (*InR*) [18] are required to promote targeting to the M6 layer. The expression of these various recognition molecules and CAMs must be tightly regulated to achieve photoreceptor subtype-specific axon terminal targeting. However, little is known about the transcriptional regulatory gene networks that lie upstream of these CAMs and recognition molecules.

A few transcription factors are known to regulate synaptic-column and layer targeting in the fly visual system. Amongst these, the zinc-finger protein Sequoia (Seq) promotes layer-specific targeting of R7 and R8 early during larval development. This is achieved by regulating the temporal expression of NCad in these photoreceptors [19]. In addition, the R8-specific transcription factor Sens has been shown to couple R8-*rhodopsin* expression with synaptic-layer targeting [20]. In the case of R7, axon projection to the M6 layer requires the repression of *sens* by the NFY-C transcription factor [20]. In the absence of *NFY-C*, *sens* is ectopically expressed in R7 and in turn, promotes the expression of *caps* and probably that of other recognition molecules [20]. This results in R7 photoreceptors projecting their terminals to the M3 layer, where R8 axons normally terminate. This phenotype is, however, not seen in all *NFY-C* mutant photoreceptors, indicating that at least one other pathway might regulate layer-specific targeting in R8.

Here, we have identified the homeobox-containing protein Otd, a transcription factor known to govern photoreceptor morphogenesis and *rhodopsin* expression [21–24], as a regulator of synaptic-column and-layer targeting in the fly retina. Otd is the founding member of the mammalian OTX1/OTX2/CRX family of transcription factors. These factors regulate brain patterning and retinal morphogenesis across phyla. In the visual cortex, OTX1 regulates the connectivity of cortical neurons with subcortical projections [25]. Moreover, in mice, *CRX*-mutant photoreceptors are unable to initiate appropriate synaptogenesis in the outer plexiform layer [26]. These findings indicate a potential role for this gene family during neural circuit development, but how these factors might achieve such a function is not understood. We show that *otd* function is required in R1-6 to promote synaptic-column specific innervation of the lamina. We find that this new function for *otd* is in part mediated by the recognition molecules Fmi and Gogo. In addition, our work demonstrates that a similar genetic network employing *otd*, *fmi*, *gogo* and the CAM *caps*, regulates the layer targeting of the R8 photoreceptor subtype. In this photoreceptor subtype, we find that layer-specific targeting is regulated by both *otd* and *sens*, which function as independent transcriptional inputs regulating a partially overlapping set of CAMs and recognition molecules.

## Results

### *otd* is required for synaptic-column targeting of R1-6 in the lamina

**Fig 2:**
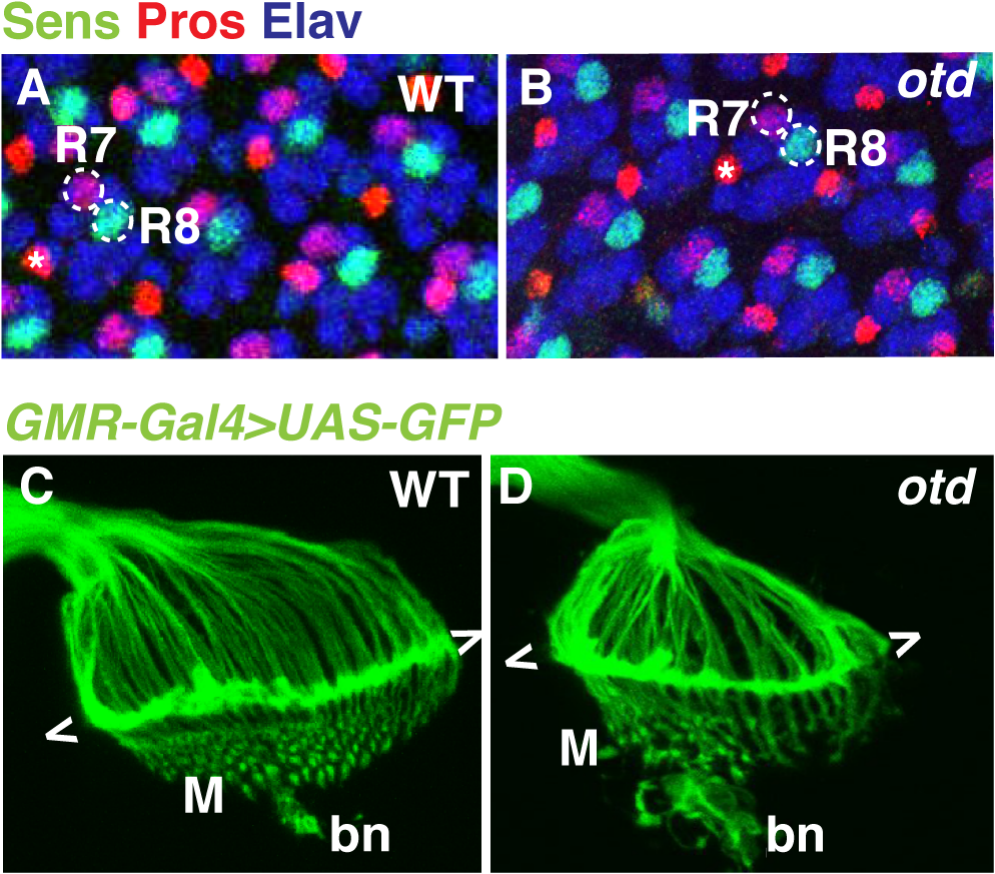
The early pattern of photoreceptor axon projections is affected in *otd* mutants. Wild-type (**A**) and *otd* ^*uvi*^ mutant (**B**) eye imaginal discs from third instar larvae stained for the neural marker Elav (blue) and for the R7- and R8-specific markers Pros (red) and Sens (green) respectively. Asterisks indicate the position of the bristle cell. Wild-type (**C**) and *otd* ^*uvi*^ (**D**) photoreceptor axon projections in third-instar larva were visualized by expressing *UAS-mCD8*::*GFP* under the control of the *GMR-Gal4* driver [43]. The growth cones of R1-R6 form a neural plexus (chevrons) at the lamina (L). R8 axons project to the medulla neuropile (M). bn stands for the Bolwig’s nerve.

*otd* is expressed in all differentiating photoreceptors, but all indications are that it is not required for cell fate specification [21,22]. This is supported by the fact that *otd* mutant ommatidia contain the full complement of photoreceptors including R1-6 and both R7 and R8 (Fig 2A and 2B).

To examine the early pattern of connectivity of *otd* mutant photoreceptors, we labelled the axons of all photoreceptors in third instar larvae by expressing *UAS-mCD8*::*GFP* under control of the *GMR-Gal4* driver. In wild-type larvae, the R1-R6 axons terminate in the lamina plexus forming a continuous line of fluorescent signal, while R8 neurons project through the lamina and form a regular array in the medulla neuropile (Fig 1A, Fig 2C). In *otd* mutant retinae, the axon projections of R1-6 terminate in the lamina but tend to form bundles (Fig 2D). In addition, the medulla, which at this early developmental stage predominantly contains R8 axons, appears disorganized (Fig 2D). These observations prompted us to further investigate the role of *otd* during photoreceptor axon targeting.

**Fig 3:**
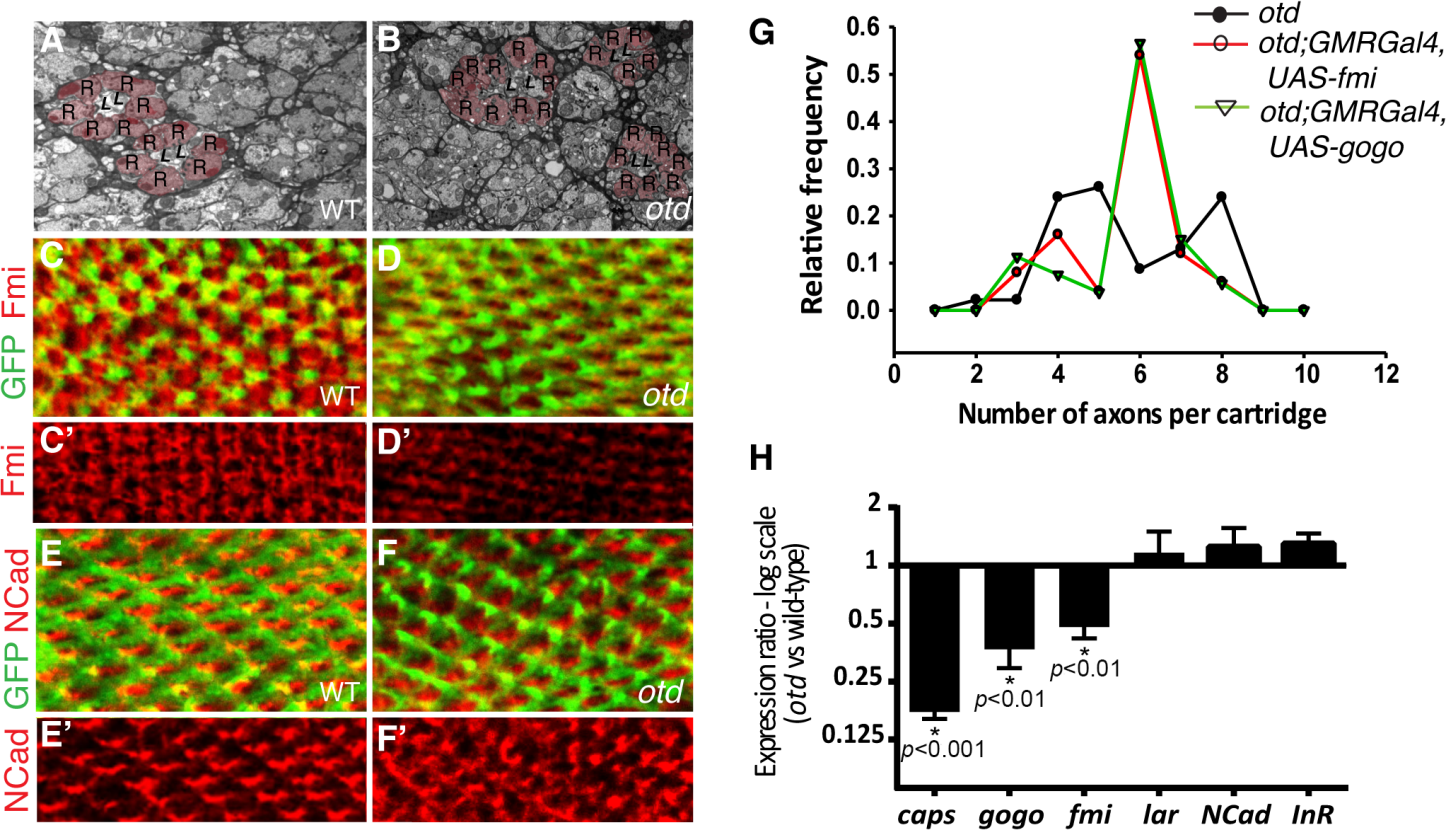
*otd* is required for synaptic-column targeting of R1-6 in the lamina. Electron micrographs of lamina cross-sections showing the normal organization of synaptic-columns in a wild-type lamina (**A**) and the defects in lamina column innervation observed in the case of *otd* mutant retina (**B**). Photoreceptor terminals are colored in pink (R), central lamina neurons are labeled ‘L’. Confocal sections of wild-type (**C-C’,E,E’**) and *otd* mutant (**D,D’,F,F’**) lamina at 40% after puparium formation. R1-6 axons are labeled by mCD8::GFP (green in **C,D** and **E,F**) and stained for Fmi (**C-D’**) (red) or NCad (**E-F’**) (red). (**G**) Frequency distribution polygon showing the numbers of R1-6 axons terminals innervating each synaptic column in *otd* ^*uvi*^ mutant lamina (black line) and in *otd* ^*uvi*^ mutants where either *fmi* (red line) or *gogo* (green line) are expressed using the GMR-Gal4 driver line. Data were gathered from EM micrographs. A Levene's test for the equality of variances, was applied. The variance in the number of axon terminals per cartridge is significantly lower in the *otd* ^*uvi*^; *GMR-Gal4; UAS-fmi* and *otd* ^*uvi*^ *; GMR-Gal4; UAS-gogo* lamina (1.66 and 1.70 respectively) compared to *otd* ^*uv*^ mutant flies (2.83, p<0.05 in both cases). (**H**) Real-time PCR quantification of *caps*, *gogo*, *fmi*, *lar*, *NCad* and *InR* mRNA normalized to *GAPDH* mRNA levels comparing wild-type and *otd* mutant retina at 40% after puparium formation. *n* = at least three independent mRNA extracts from wild-type and *otd-*mutant retinas. Error bars represent SEM.

We first examined the lamina column targeting of *otd* mutant R1-6 photoreceptors in more detail. To this end, we used electron microscopy to quantify the number of afferent R1-6 photoreceptor neurons that target each lamina column (also referred to as lamina cartridge). In the main part of the retina, outside of the midline of wild-type eyes, six R1-6 afferent axons converge on one synaptic column in the lamina (Fig 1B and 3A). In contrast, we find that in the case of retinae mutant for *otd*, the number of axon terminals that innervate the lamina columns varies, ranging from 2 and 10 (Fig 3B and 3G). This quantification reveals a failure of the R1-6 axons to innervate their appropriate synaptic-columns, demonstrating that *otd* is required for axonal targeting of R1-6 in the lamina.

### *fmi* and *gogo* function downstream of *otd* in R1-6

The photoreceptor targeting phenotype observed in the larval optic lobe and in the adult lamina in the absence of *otd* is reminiscent of that described in photoreceptors mutant for the recognition molecules and CAMs *fmi*, *gogo* or *NCad* [12,14,16]. During synaptic-column targeting in the lamina, these proteins promote interactions between R1-6 growth cones within each fascicle. This drives each growth cone to separate from the fascicle and extend laterally, to innervate the appropriate synaptic- column target [27,28]. Consistent with a function for *otd* in regulating appropriate R1-6 targeting in the lamina, we find that, when compared to wild-type (Fig 3C and 3C’), the protein expression of Fmi is greatly diminished in the growth cones of *otd* mutant R1-6 (Fig 3D and 3D’). In addition, a significant reduction of both *fmi* and *gogo* mRNA levels can be measured in developing retina mutant for *otd* (Fig 3H). Whereas the mRNA and protein levels of *NCad (* Fig 3E, 3F’ and 3H), as well as the mRNA levels of *LAR* (Fig 3H) which both regulate column targeting of R1-6 [14,15], remain unchanged.

Our data suggest that *otd* is required for sorting the R1-6 axons into their respective lamina columns *via* promoting the expression of *fmi* and *gogo*. Relative differences in the expression levels of Fmi between R1-6 growth cones within the same fascicle regulate neural superposition establishment [29]. Overexpressing *fmi* in R1-6, using the GMR-Gal4 leads to high levels of Fmi in R1-6 growth cones (S2 Fig) and is accompanied by defects in neural cartridges morphology including variable numbers of R1-6 terminal inputs (S2 Fig). However, we also observe numerous wild-type neural cartridges, indicating that the GMR-Gal4 driver line might be suitable for attempting rescue experiments using the *otd* ^*uvi*^ mutant and the *UAS*-*fmi* strains.

Therefore, to test the hypothesis that *fmi* and *gogo* mediates part of *otd* function during neural superposition, we used electron microscopy to quantify the number of afferent *otd*-mutant R1-6 axons in lamina columns in which either *fmi* or *gogo* was re-introduced using the GMR-Gal4 driver line. Our quantification revealed that re-introducing *fmi* or *gogo* in *otd*-mutant photoreceptors partially restores the distribution of R1-6 axon projections towards the wild-type situation of six afferent terminals per synaptic-column (Fig 3G). We note that while the overall levels of Fmi are higher in *otd* ^*uvi*^ mutant R1-6 when re-introducing Fmi using the GMR-Gal4 driver, R1-6 growth cones still exhibit differences in Fmi levels (S2 Fig). Taken together, our data demonstrate that *otd*-dependent transcriptional regulation of the recognition molecules *fmi* and *gogo* is required for the proper synaptic-column targeting of R1-R6 in the developing lamina.

### *otd* is required for synaptic-layer specific targeting of R8

**Fig 4:**
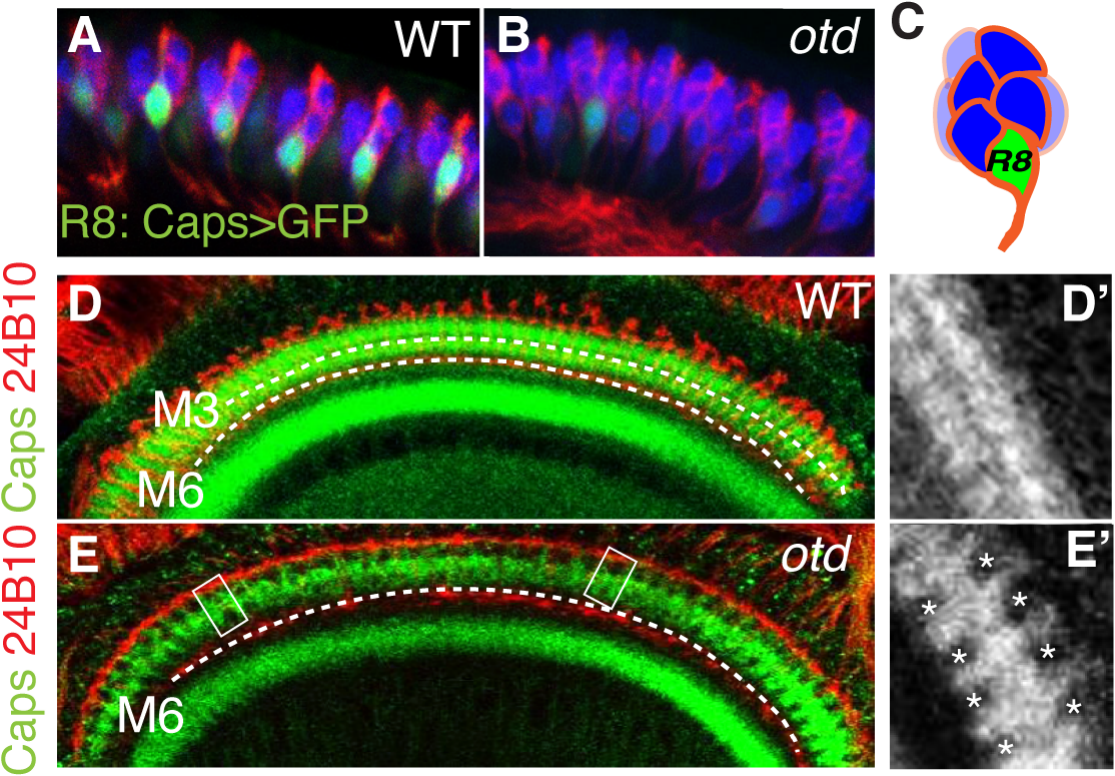
Caps expression is downregulated in *otd* ^*uvi*^ mutant R8 photoreceptors. Side view of wild-type (**A**) and *otd* ^*uvi*^ mutant (**B**) third instar larva eye disc revealing that *caps* expression is strongly reduced in *otd* mutant R8. The expression of mCD8-GFP is under the control of *caps-Gal4 [8]*. Elav (blue) and 24B10 (red) stain the full set of photoreceptors. (**C**) Representation of an ommatidium showing the basal localization of the R8 photoreceptor in green. Expression of Caps (green) in wild-type (**D, D’**) and *otd* ^*uvi*^ mutant (**E, E’**) optic lobes (60% after puparium formation). Photoreceptor-cell-axons are stained with the 24B10 antibody. The R8 (M3) and R7 (M6) recipient layers are indicated by dashed lines in this and the following figures. When compared to wild-type, *otd* ^*uvi*^ mutant retina show a reproducible gap pattern detected in the R8 layer (M3) (**D’, E’**). These gaps correspond to a loss of Caps expression in the afferent R8 axons (boxed in **E** and magnified in **E’**).

Processing of chromatic information relies on R7 and R8 establishing their synapses in two distinct layers of the medulla (Fig 1B and S1 Fig). In R8, layer-specific targeting relies on the expression of *fmi*, *gogo* and *caps*, amongst other adhesion and recognition molecules. Interestingly, similar to *fmi* and *gogo*, we find that the mRNA levels for *caps* are greatly reduced in *otd* mutant retina (Fig 3H). This reduction is also observed in R8 using an *in vivo* reporter gene for the *caps* locus, *capsGal4-UASGFP* [8] (Fig 4A and 4B). In addition, the Caps protein is no longer detected in the *otd* mutant R8 axons, leading to gaps in expression at the M3 layer, where the R8 axons normally terminate (Fig 4D and 4E’). Therefore, *otd* is required for the expression of key recognition molecules and CAMs that are known to govern the layer-specific targeting of R8.

**Fig 5:**
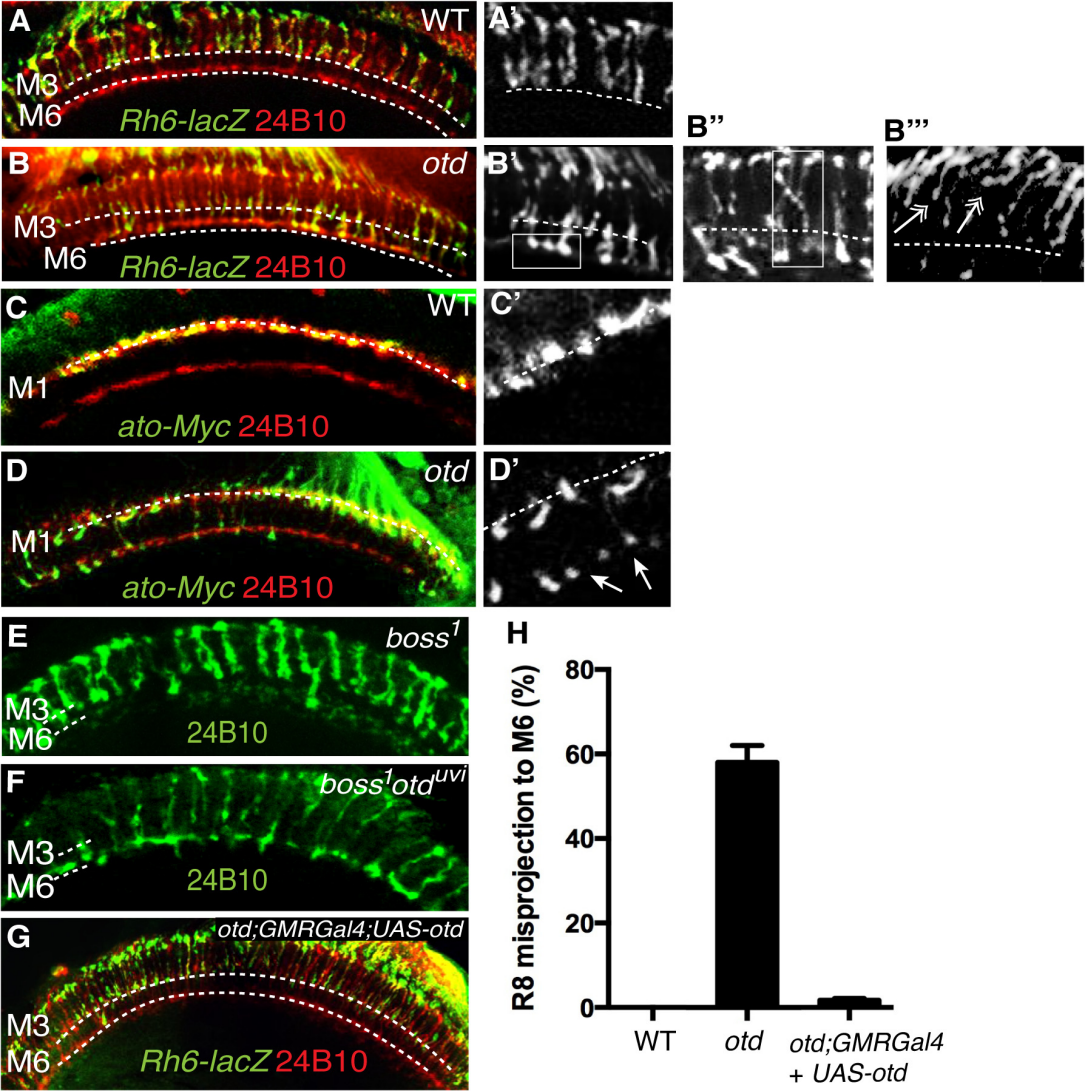
*otd* is required for R8 synaptic-layer targeting. Wild-type (**A,A’**) and *otd* ^*uvi*^ mutant (**B,B’**) adult optic lobes expressing the R8 specific marker *Rh6-lacZ* stained with anti-β-galactosidase (green). In wild-type (**A,A’**) Rh6-lacZ-positive R8 axons terminate in the M3 layer. *otd* ^*uvi*^ mutants (**B-B”**) display a strong R8 axon misprojection phenotype. R8 axons specifically overshoot to the M6 layer (boxed in **B’** and quantified in Fig 5H) while some R8 axons invade neighbouring columns cross-laterally and terminate in abnormal positions (boxed in **B”**). Several R8 axons terminals can also be seen to stall at more superficial layers and fail to innervate the medulla (double arrows in b”‘). All panels (**C-F**) show photoreceptor axon projections stained with the 24B10 antibody (red). R8 axons in wild-type (**c,c’**) and *otd* ^*uvi*^ mutant (**D,D’**) optic lobes (40% after puparium formation) are visualized using the *ato-τ-myc* transgene stained with anti-Myc antibody (green). At this early stage of development, in *otd* ^*uvi*^ mutant retina, R8 growth cones fail to stop in the M1 layer and extend specifically to the R7 temporary layer (n = 438 misprojecting axons of 798 R8 axons quantified in the two temporary layers). The staining for ato-*τ*-Myc is magnified in (**C’,D’**) and arrows in (**D’**) indicate misprojecting R8 axons. *boss* ^*1*^ mutant (**E**) and *otd* ^*uvi*^ */boss* ^*1*^ double-mutant (**F**) retina. Adult optic lobes stained with 24B10 antibody. In (**E**), residual 24B10 staining in the R7 M6 layer is derived from medulla neurons [44]. (**E**) *boss* ^*1*^ mutant R8 terminate in the R8 recipient layer M3, with only a few R8 targeting to the M6 layer. (**F**) Most of the *otd* ^*uvi*^ */boss* ^*1*^ double-mutants display a strong R8 mis-projection phenotype in the R7 layer, M6. (**G**) *otd* ^*uvi*^ mutant flies where Otd expression has been restored specifically in the photoreceptors using the *GMRGal4* driver. In this context, staining of the R8 specific marker *Rh6-lacZ* (green) demonstrates that normal R8 photoreceptor targeting is almost completely restored. (**H**) Quantification of the misprojections of *Rh6-lacZ*-positive R8 axons in wild-type, *otd* ^*uvi*^ mutant and *otd* ^*uvi*^ mutant flies where Otd expression has been restored using the *GMR-Gal4* driver.

These experiments suggest that in the absence of *otd*, layer-specific targeting of R8 should be affected. To test this hypothesis, we examined the projection pattern of R8 terminals in *otd* ^*uvi*^ animals, using the R8 specific *Rh6-lacZ* reporter transgene. When examining adult medulla stained for *Rh6-lacZ*, we found that 59% (n = 503 of 854) of mature R8 terminals fail to stop in the M3 layer and instead terminate in the M6 layer, where R7 terminals are normally found (Fig 5A, 5B’ and 5H). This phenotype can also be detected using the null allele *otd* ^*JA101*^ [30] combined to the MARCM system [31] (S3 Fig). In this case, as for *otd* ^*uvi*^, not all R8 axons project ectopically in the M6 layer, suggesting that during R8 synaptic layer-specific projection, another redundant pathway might function in parallel of *otd*.

In addition to the ectopic projection of R8 terminals to the M6 layer, we also detect various defects that include crossing of R8 terminals into neighbouring columns and column thickening (Fig 5B”). These phenotypes are reminiscent of those originally reported for *caps* mutants [8]. In addition, several R8 terminals are stalled in a more superficial layer, at the periphery of the medulla. This phenotype is difficult to quantify but is readily visible in medulla stained for the R8 reporter Rh6-LacZ (Fig 5B”‘). This stalling phenotype is similar to that described for the *fmi* and *gogo* mutant R8s [17]. Thus, taken together, our data are consistent with *otd* being required for the expression of *fmi*, *gogo* and *caps* in R8.

Interestingly, when we tested for axon projection defects at early developmental stages, before R7 and R8 have reached their final recipient layers (M6 and M3 respectively), we found that up to 55% (n = 438 of 798) of *otd* mutant R8 terminals already misproject in the R7 temporary layer (Fig 5C and 5D’). This early phenotype cannot be explained solely by the loss *caps*, as the *caps* mutant phenotype has been shown to be very mild at comparable early developmental stages [8]. Given the specific early redirection of the R8 terminals to the temporary R7 layer phenotype, we conclude that in addition to being required in R8 for the expression of R8 CAMs, *otd* must also have a role in preventing R8s from becoming competent to project to the R7 layer.

In principle, the early mistargeting of R8 axons could result from the conversion of R8 photoreceptors to an R7 cell fate. To address this issue, we stained *otd*-mutant eye discs for the R7- and R8-specific markers Prospero (Pros) and Sens respectively and could confirm that no R8 to R7 transformations take place in the absence of *otd* (Fig 2A and 2B). Moreover, the expression of *sens* is maintained in R8 during pupal development (S4 Fig). This finding excludes the possibility that *otd* indirectly affects R8 axon targeting by regulating cell fate specification. Alternatively, as the majority of *otd*-mutant R8 axons specifically innervate the R7 layer, this raises the possibility that they might be unable to defasciculate from their R7 partner. To investigate this possibility, we tested whether R7 neurons contribute to the *otd* mutant R8 mistargeting phenotype by looking at *boss* ^*1*^ mutant ommatidia, which lack the R7 photoreceptor. In *otd*/*boss* ^*1*^ double mutant flies, we found that the *otd* mutant R8 mistargeting to the M6 layer is maintained (60% n = 149 of 251). This indicates that the *otd* phenotype does not depend on the presence of the R7 axon (Fig 5E and 5F).

Finally, we sought to test if the *otd* mutant R8 photoreceptors that misproject to the M6 layer are able to form synapses in this layer. To this end, we used an antibody raised against Bruchpilot, a protein localized to the active zone of synapses of the optic neuropile [32,33]. We found that Bruchpilot staining co-localized with the terminals of R8 photoreceptors that project ectopically in the M6 layer (S5 Fig). Given the crowded nature of the medulla, in which many different cell types must specifically interconnect at every layer, some nonspecific co-localization of antibody staining might occur. To rule out this possibility, we used an R8-Gal4 line to specifically express a GFP-labeled version of Bruchpilot (UAS-BRP-GFP) in R8 neurons, thereby visualizing only those synapses made by R8 axons (S5 Fig). The co-localization between the GFP signal and R8 terminals indicates that *otd* R8 axons that misproject to the M6 layer are likely to establish synaptic connections, within this layer. Therefore, while *otd* is required for the expression of *fmi*, *gogo* and *caps* in R8, it seems to be dispensable for proper synaptogenesis.

### *otd* functions in the R8 photoreceptor

Most of the experiments described thus far were carried out using an eye specific *otd* allele, *otd* ^*uvi*^. This allele consists of a partial deletion of an eye-specific enhancer and results in a strong reduction in Otd protein expression in photoreceptors [21]. To ensure that the *otd* phenotype we report here is due to a function of *otd* in photoreceptors and not in the developing optic lobe, we used three distinct approaches. First, to evaluate the possibility of a reduction in *otd* expression in *otd* ^*uvi*^ brain, we assessed *otd* mRNA levels in the optic lobes of wild-type and *otd* ^*uvi*^ flies. We found no difference in *otd* transcript levels between wild-type and *otd* ^*uvi*^ mutants in the optic lobe, whereas, in the retina of *otd* ^*uvi*^ flies, *otd* expression is downregulated by approximately 50-fold compared to wild-type (S4 Fig). Second, we induced eye-specific RNAi-mediated knockdown of *otd* expression using two different *UAS-otd* RNAi lines combined with the eye specific *GMR-Gal4* driver line. These two lines are effective at knocking down *otd* expression in the photoreceptors (S4 Fig). The specific R8 mistargeting phenotype characteristic of *otd* was seen with both of these RNAi lines (S4 Fig). The misprojection phenotype was less severe in the *otd* RNAi knockdown lines (24% n = 115 of 479) than in *otd* ^*uvi*^. This correlates with the residual expression levels seen in knockdown lines (S4 Fig) and suggests that the penetrance of the *otd* mutant phenotype in R8 depends on the transcript level. Third, we performed photoreceptor-specific rescue experiments using the *GMR-Gal4* driver and available *UAS-otd* transgene lines to re-introduce *otd* specifically in the retina in *otd* ^*uvi*^ mutant flies. With this approach, the R8 synaptic-layer targeting defects quantified in *otd* ^*uvi*^ mutant retinae can be almost completely rescued. R8 misprojections in the rescued flies were found to be 2% (n = 9 of 451), compared to the 59% misprojection seen in the *otd* ^*uvi*^ mutant (Fig 5G and 5H). In these retinas, the *caps* mRNA is restored to levels that are comparable to those measured in wild-type retina (S6 Fig). In addition, the levels of *fmi* and *gogo* mRNA are also significantly increased and thus closer to wild-type when compared to *otd*-mutant retina (S6 Fig). Importantly, overexpressing *otd* in an otherwise wild-type retina does not affect the pattern of projection of R7 and R8 (S7 Fig). Taken together, our data demonstrate that the requirement of *otd* for the expression of optimal levels of *caps*, *fmi* and *gogo* is autonomous to the R8 photoreceptor.

### *otd* and *sens* provide independent transcriptional inputs during R8 layer targeting

**Fig 6:**
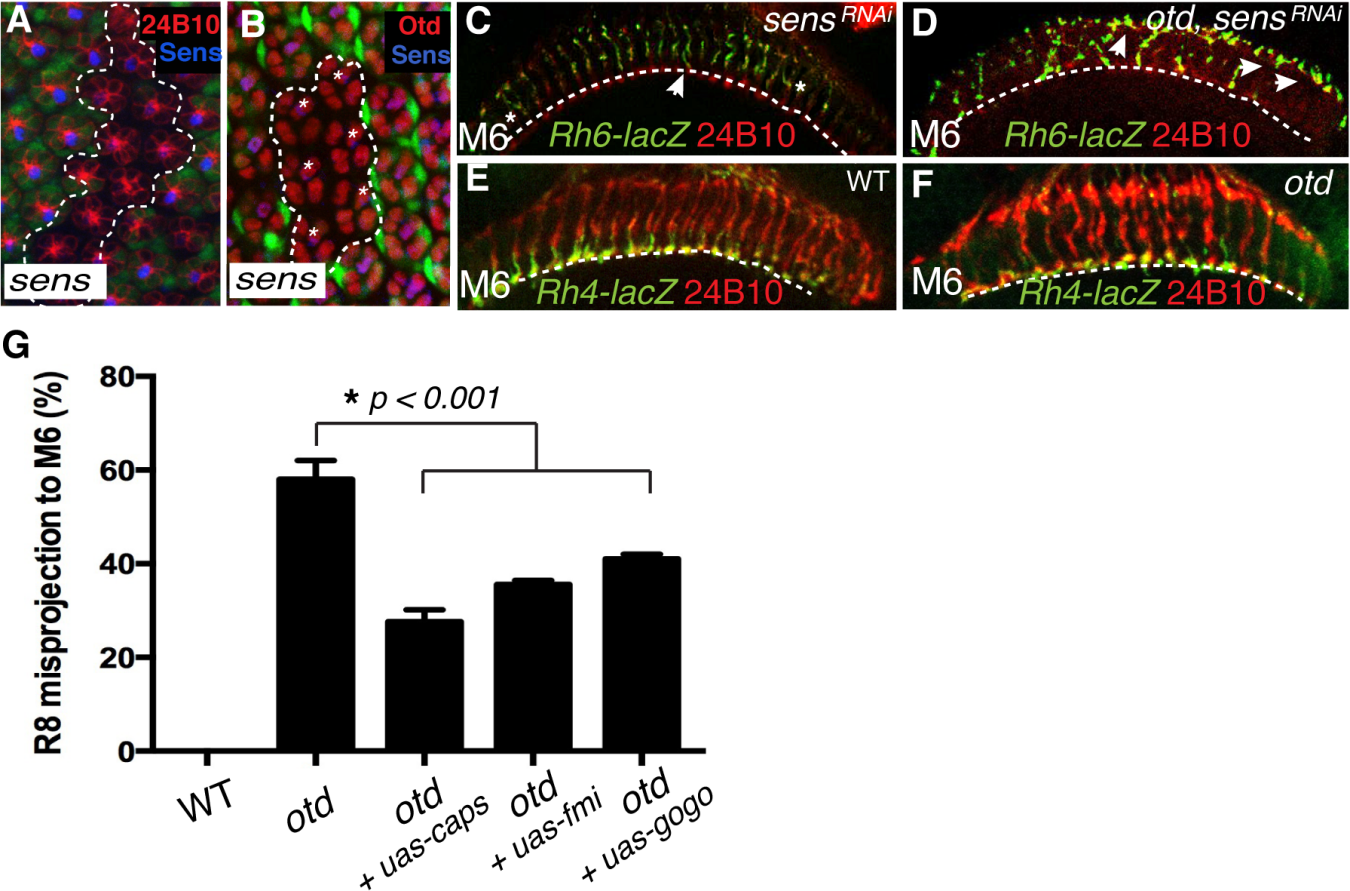
*otd* and *sens* are required for R8 synaptic layer targeting. All panels (**A-F**) show photoreceptor cell projections stained with 24B10 (red). (**A**) Expression of *UAS-sens* ^*RNAi*^ (GFP-negative ommatidia, encircled by a dotted line) in wild-type tissue (GFP positive) 48 h after clone induction using the tub>GFP>Gal4 system. *UAS-sens* ^*RNAi*^ expressing cells show a clear reduction in Sens protein levels (blue). (**B**) Expression of *UAS-sens* ^*RNAi*^ transgene (GFP-negative ommatidia, encircled by a dotted line) in wild-type tissue (GFP positive) 48 h after clone induction using the tub>GFP>Gal4 system. Otd expression (red) is unaffected in *UAS-sens* ^*RNAi*^ expressing R8 cells (indicated by asterisks). (**C**) Rh6-lacZ-positive R8 axons in *sens* ^*RNAi*^ (R8-specific driver*-Gal4* ^*109–68*^;*UAS*-*sens* ^*RNAi*^) and in *otd* ^*uvi*^ mutant combined with *sens* ^*RNAi*^ retina (**D**), stained with anti-β-galactosidase (green). *sens* knockdown in the retina leads to a few defects in R8 axon projection, however the combination of the *sens* knockdown and *otd* ^*uvi*^ mutant leads to a complete failure of R8 layer-specific targeting, indicating that *sens* and *otd* act in parallel. (**E,F**) Layer-specific targeting of the R7 photoreceptors is assessed using the R7 specific transgene *Rh4-lacZ*. As in wild-type (**E**), Rh4-lacZ positive R7 terminals (green) correctly target to the M6 layer in *otd* ^*uvi*^ mutants flies (**F**). (**G**) Quantification of the misprojections of *Rh6-lacZ*-positive R8 axons in wild-type, *otd* ^*uvi*^ mutant and *otd* ^*uvi*^ mutant flies where either *caps*, *fmi* or *gogo* expression has been restored. Since Caps is present only in R8, *UAS*-*caps* is expressed under the control of an R8-specific Gal4 driver, while *UAS*-*gogo* and *UAS*-*fmi are* expressed using the pan-photoreceptor *GMR-Gal4* driver. Restoring *caps* expression in *otd*-mutant R8 photoreceptors partially rescues the *otd* ^*uvi*^ mutant R8 mis-targeting phenotype (from 59% R8 misprojection in *otd* ^*uvi*^ to 25% in *otd* ^*uvi*^ *+ UAS-caps*). Similarly, expression of *fmi* and *gogo* in *otd* ^*uvi*^ *mutant* photoreceptors partially suppress the R8 misprojection phenotype (36% R8 axons misprojecting in *otd* ^*uvi*^ *+ UAS-fmi*, n = 374 of 1039; 40% R8 axons misprojecting in *otd* ^*uvi*^ *+ UAS-gogo*, n = 436 of 1090). Percentages indicate quantitative assessment of R8 axons as detected in M3 and M6 layers only. In wild-type, all R8 neurons target to the M3 layer, thus the mistargeting percentage is zero.

The *otd* mutant defects in R8 layer targeting described here are also observed in R8s that lack the gene encoding the transcription factor *sens* [20]. Thus, our data raise the possibility that *sens* might be downregulated in the absence of *otd*. When we tested this hypothesis, we found this not to be the case, as *sens* is expressed normally in the absence of *otd* (Fig 2B). Likewise, *otd* expression is unaffected in photoreceptors in which *sens* expression has been reduced (Fig 6B). Therefore, *otd* must function in parallel to *sens* during layer targeting of R8 photoreceptors.

If this model is correct, attenuating the expression of both *otd* and *sens* should lead to a complete failure for R8 terminals to reach the M6 layer. To test this model we generated *otd* mutant photoreceptors in which the *sens* transcript was reduced using a specific RNAi strain (tested in Fig 6A), thus still allowing for normal R8 specification [34]. Consistent with our model, we observed a complete failure of R8 layer targeting in the medulla in the double mutant *otd* ^*uvi*^ */sens* ^*RNAi*^ flies with a majority of R8 terminals either stalling at a more superficial layer or overshooting specifically to the M6 layer (Fig 6D). This demonstrates that *otd* and *sens* provide independent transcriptional inputs during synaptic-layer targeting of the R8 photoreceptor.

### *caps*, *fmi* and *gogo* function downstream of *otd* in *R8*

Next, in order to test if *caps*, *fmi* and *gogo* function downstream of *otd* during R8 layer targeting, we asked if re-introducing these genes in an *otd* ^*uvi*^ mutant retina suppresses the R8 mis-targeting phenotype. To this end, we used both a pan-photoreceptor and an R8 specific Gal4 strain combined with either *UAS-fmi [35]*, *UAS-gogo* [16] or *UAS-caps* [8]. The *Rh6-LacZ* transgene was used to score the R8 axon terminals in the medulla. Re-introducing *caps* leads to a partial rescue of the R8 mis-targeting phenotype (from 59% R8 mis-targeting in *otd* ^*uvi*^ to 25% in *otd* ^*uvi*^ *+ UAS*-*caps*; n = 225 of 903). *fmi* and *gogo* cooperate in a non-redundant manner during R8 layer targeting [17]. The expression of these two recognition molecules is not completely abolished in *otd* ^*uvi*^ mutant retinae and is instead likely to be sub-optimal (Fig 3D’ and 3H). In this context, re-introducing *fmi* or *gogo* gives a partial rescue of the R8 mis-targeting phenotype (36% of R8 axons mis-target in *otd* ^*uvi*^ *+ UAS*-*fmi*, n = 374 of 1039; 40% of R8 axons mis-target in *otd* ^*uvi*^ *+ UAS*-*gogo*, n = 436 of 1090) (Fig 6G). We attribute this partial rescue to the suppression of the stalling phenotype of R8 terminals (Fig 5B”‘) thereby resulting in a greater proportion of *otd* mutant R8 axons projecting correctly to the R8 layer. Altogether, these experiments demonstrate that *otd* is required for the synaptic-layer targeting of R8, at least in part by promoting the expression of *fmi*, *gogo* and *caps*.

### *otd* is not required for synaptic-layer targeting of R7

As *otd* is expressed in all photoreceptors, it might also be required for the projection of R7 photoreceptors. To address this question, we examined the layer-specific targeting of the R7 photoreceptors *in vivo* using the R7 *Rh4-lacZ* reporter. We found that all *otd* mutant R7 axons stop correctly in the M6 layer (Fig 6E and 6F). Thus *otd* function during column- and layer-specific targeting is restricted to the R1-6 and R8 photoreceptor subtypes. Consistent with a lack of phenotype during R7 layer-specific targeting, we found that the expression levels of the main recognition molecules that govern this process (i.e., *NCad*, *LAR* and *InR*) are not affected in the absence of *otd* (Fig 3H).

## Discussion

**Fig 7:**
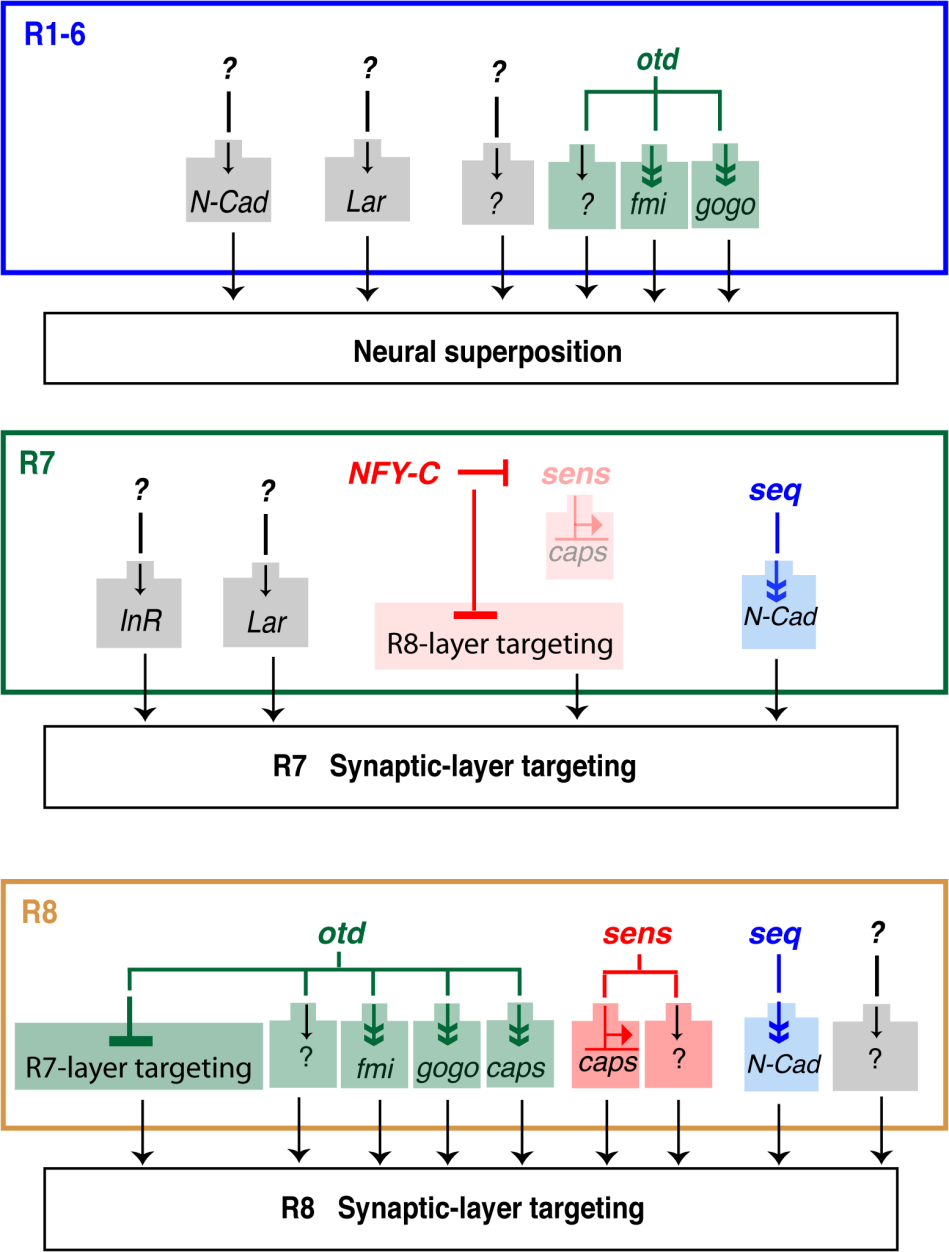
Transcriptional inputs required for proper photoreceptor targeting in the *Drosophila* visual system. Summary of the transcription factors that regulate column- and layer-specific targeting of the R1-6 and R7 and R8 photoreceptors. Predicted indirect regulation is shown with a double arrowhead (Sens binds directly on the *caps* promoter). Although all of the currently known transcriptional regulators (i.e., *seq*, *sens* and *otd*) are presented, for clarity, only those CAMs and adhesion molecules most relevant to our findings are shown here.

Little is known about the transcriptional regulation that controls the establishment of genetically hardwired patterns of synaptic connections during development. The factors and molecular pathways that govern the establishment of the typical pattern of R1-6 connectivity in the lamina have yet to be fully characterized. During neural superposition in the main part of the retina, each lamina column is innervated by axons coming from at least six neighbouring facets (i.e., one axon per facet is contributed to one synaptic-column) (Fig 1B). Although the CAMs and recognition molecules NCad, Fmi, Gogo and LAR have been shown to regulate this process, very little is known about the upstream transcriptional regulatory gene networks that control the expression of these molecules. Here we identify *otd* as a transcription factor required for synaptic-column targeting of R1-6 in the fly visual system (Fig 7). We show that in the developing retina, this phenotype correlates with a significant downregulation of *fmi* and *gogo*, and that re-introducing these genes in *otd* mutant R1-6 can ameliorate the lamina column innervation defects observed in *otd* ^*uvi*^ mutant retinae. This indicates that *otd* function is required in R1-6 to promote the expression of optimal levels of these key factors to control lamina synaptic-column targeting. Our finding that the expression of NCad or LAR are not affected by the loss of *otd* suggests that additional independent transcriptional inputs are likely to operate in R1-6 during synaptic-column innervation.

Within each axon bundle, relative differences in Fmi expression promotes the lateral extension of the R1-6 growth cones so that they project toward different neural cartridges [12,29]. In this context, Fmi and N-Cad act redundantly [36]. Interestingly, it is not clear how *fmi* expression is differentially regulated in R1-6. One possibility is that expression of *fmi* is solely regulated at the transcriptional level. Another possibility is that differential expression of Fmi between R1-6 growth cones involves both transcriptional and posttranscriptional regulations. Alternatively, as photoreceptors are recruited sequentially during retina morphogenesis, differences between R1-6 expression levels might also be due to the onset of transcription at the *fmi* locus. Our ability to ameliorate the *otd* ^*uvi*^ phenotype in the lamina by re-introducing *fmi* (or *gogo*), suggests that post-transcriptional regulation of these molecules might operate during neural superposition. As expected, when we express *fmi* using the GMR-Gal4 driver line in otherwise *otd* ^*uvi*^ mutant animals, we observe an overall increase in the level of the Fmi protein in the corresponding lamina. However, we note that the Fmi staining remains relatively heterogeneous, and reminiscent of the Fmi staining seen in wild type lamina. While this suggests a level of post-transcriptional regulation it might equally be explained by the presence of another factor, present at different levels in each R1-6 cells, and that might limit the concentration Fmi at the growth cone. It is also possible that the temporal pattern of expression of the *GMR-Gal4* driver line is compatible with the endogenous pattern of *fmi* expression. In favour of this notion, although overexpressing *fmi* with this driver line in an otherwise wild-type retina leads to defects in neural superposition, we also detect many wild-type lamina cartridges, despite the fact that in these experiments the overall levels of Fmi are higher than in wild-type. Further work will be required to understand how exactly the expression of Fmi is regulated at the level of the R1-6 growth cones during neural superposition.

In addition to being required for proper synaptic-column targeting of R1-6, we demonstrate that *otd* is specifically required for layer-specific targeting of the R8 photoreceptor subtype (Fig 7). During early phases of visual system development, dynamic expression of *seq* in R7/R8 regulates the expression of *NCad* to ensure the separation of the R7 and R8 terminals in their respective temporary layers [19]. Later in development, *sens* has been shown to coordinate the expression of *rhodopsins* [20,37] and layer-specific targeting in R8. One of the CAMs implicated downstream of *sens* during R8 synaptic-layer targeting is *caps* [20]. In *sens-*mutant R8, a fraction of R8 terminals project ectopically to the M6 layer (where R7 terminals normally project). Interestingly, in *NFY-C* mutant R7 photoreceptors, where *sens* and *caps* are ectopically expressed, not all terminals misproject in the M3 layer (where R8 terminals are normally found). Taken together, this indicates that at least one other pathway must be involved in regulating R8 layer-specific targeting. Our work indicates that *otd* regulates this process independently of *sens* and thus is part of a new gene regulatory network that cooperates with *sens* during R8 layer targeting. Our finding that the expression of *otd* and *sens* is not interdependent suggests a model in which these two transcription factors act in parallel pathways in R8. Because *sens/otd* double mutant R8s show an additive phenotype when compared to the respective single mutants, we conclude that *otd* and *sens* provide convergent transcriptional inputs during R8 synaptic-layer targeting.

In R8, we find that *otd* function correlates with a transcriptional downregulation of *fmi*, *gogo*, and *caps*. *fmi* or *gogo* mutant R8 are characterized by strong axon targeting defects. R8 axons form bundles and often stall at the surface of the medulla in the temporary M1 layer [7,12,16]. This is thought to be due to a function of these two factors in promoting the release of the R8 growth cones from the M1 temporary layer, thus allowing them to innervate their final destination, the M3 synaptic-layer. *caps* function in R8 is a little more controversial. Early work indicates that it is important for the proper stabilization of R8 terminals in the M3 layer in the medulla [8]. However, recent work indicates that *caps* function in R8 might be much more limited than previously thought and that perhaps other members of the leucine-rich-repeat gene family might act redundantly with *caps* during R8 layer targeting [38]. Nevertheless, ectopic *caps* expression in R7 is sufficient to re-direct their axon terminals to the M3 layer where R8 normally terminates [8].

The range of R8 projection phenotypes that we observe in *otd* mutant retinae is compatible with *fmi*, *gogo* and *caps* mediating at least part of *otd* function during R8 synaptic-layer targeting. However, our work demonstrates that even early in visual system development (i.e. 40% APF), a large proportion of the *otd* mutant R8 axons project their terminals specifically to the R7 temporary layer. This early misprojection phenotype for the R8 axon terminals is not seen in the *fmi* or *gogo* single mutants. In addition, this phenotype is only detected at a very low frequency in *caps* mutants [8]. Therefore, the phenotype caused by loss of *otd* function in R8 cannot be fully explained by the measured decrease in *caps*, *fmi* and *gogo* expression. One possible explanation for the early misprojection of R8 terminals to the R7 temporary layer is that *otd* might be required in R8 to suppress at least part of the R7-specific genetic program that governs axon terminal projection to the R7 synaptic layer. This scenario would be analogous to the situation found in the R7 subtype, where *NFY-C* represses part of the R8-targeting genetic network [20]. The genetic program that governs axon projection in R7 is not fully understood. Further work will therefore be required to test the hypothesis that *otd* is required in R8 to prevent these neurons from becoming competent to project to the M6 synaptic layer where R7s normally project.

Nevertheless, we present evidence that *fmi*, *gogo* and *caps* act downstream of *otd* in R8. Given that ectopic Caps expression in R7 is sufficient to redirect R7 terminals to the R8 M3 layer, our ability to partially rescue the *otd*-mutant R8 misprojection phenotype by re-expressing *caps* is expected. The mild rescue we observe when re-introducing *fmi* or *gogo* is perhaps more surprising, given that these molecules function together in R8. However, although reduced, the expression of *fmi* and *gogo* is not completely lost in the absence of *otd*. Therefore, basal levels of *fmi* and *gogo* are available to support the partial rescues we obtain with *fmi* or *gogo*. Importantly, the disorganization of the R8 pattern of axon projections that we observe in the third instar larvae, together with the many instances of R8 terminals stalling at the surface of the medulla later during development, is also observed in the case of *fmi* and *gogo* single mutant R8s [16,17]. We therefore attribute the mild rescue of the *otd* mutant phenotype by *fmi* and *gogo* to a rescue of a fraction of the stalled R8 growth cones.

While our work demonstrates that *otd* is part of a new gene regulatory network required for synaptic-column and layer targeting in the fly visual system, the relationship between this transcription factor and the downstream effectors *fmi*, *gogo* and *caps* is not clear. In this context, it is interesting to note that Sens can bind to a putative Sens-binding domain in the *caps* promoter and, together with Otd, can also bind to the *rh5* and *rh6* promoters [20,37]. This raises the issue that Otd might bind and cooperate with Sens directly on the *caps* promoter and perhaps that of *fmi* and *gogo*. The promoter and potential regulatory elements of *caps*, *fmi* and *gogo* are relatively large and not well defined, making it difficult to identify potential *otd* binding sites, a tandem repeat of (TAATCC) [23]. No clear Otd binding site can be found within the *caps* promoter and in particular in the immediate vicinity of the putative Sens binding site [20]. As for the *caps* locus, examining the *fmi* and *gogo* promoters does not reveal any clear Otd binding consensus sites. In addition, cloning of a 1.5 Kb caps promoter that contains the putative Sens binding site in front of the LacZ reporter gene does not lead to any detectable expression *in vivo*. Overall, the available evidence does not support a simple and direct binding of *otd* on the *fmi*, *gogo* or *caps* promoters. Although it is not possible to rule out that Otd might bind to more distal regions of these promoters or in introns, we favor a model in which Otd regulates the expression of these recognition molecules through an indirect mechanism and/or through a complex interaction with partner transcription factors.

Taken together, our data suggest that Otd is part of a novel gene regulatory network that is required for the optimal expression of a set of conserved CAMs and recognition molecules in the developing fly visual system. Further work will be required in order to identify all of the components of this gene regulatory network. It will also be interesting to test to what extent OTX1 and OTX2, the mammalian orthologs of Otd, regulate column- and layer-specific targeting of subsets of neurons during vertebrate brain development. During brain development in mice, *otx2* has been shown to be required for the expression of the adhesion molecule RCadherin and Ephri-A2 [39]. In the chick, *otx2* is co-expressed with the *caps* ortholog leucine-rich repeat neuronal 1 (Lrrn1) at the posterior midbrain border [40]. These observations suggest that Otd might be closely associated with the expression of adhesion and recognition molecules in developing neurons across phyla.

## Materials and Methods

### Drosophila genetics

The following fly strains were used: wild-type (Canton-S). *w*, *otd* ^*uvi*^, *w*, *otd* ^*uvi*^ *; GMR-Gal4;Rh6-lacZ*, *GMR-Gal4;Rh6-lacZ*, *otd* ^*uvi*^ *;GMR-Gal4;Rh4-lacZ*, *GMR-Gal4;Rh4-lacZ*, *UAS-mCD8*::*GFP*, *UAS-sens* ^*RNAi*^ (line 27287, Bloomington Stock Center), *UAS-otd* ^*RNAi*^ (lines 29342 and 34327, Bloomington Stock Center), *otd* ^*uvi*^ *;capsGal4*, *capsGal4*, *otd* ^*uvi*^ *;GMRGal4* combined to *UAS-fmi or UAS-gogo or UAS-otd*, *the R8-specific driver Gal4* ^*109–68*^ *combined with UAS-caps-Ia5*, *ato-τ-myc*, *otd* ^*uvi*^ *; ato-τ-myc*, *boss* ^*1*^ *Sb/boss* ^*1*^ *TM6B* (gift from H. Kramer, University of Texas Southwestern Medical Center, Dallas, TX), *w*, *otd* ^*uvi*^ *; boss* ^*1*^ *Sb/boss* ^*1*^ *TM6B*; UAS-bruchpilotGFP (lines 36291, 36292, Bloomington Stock Center), *otd* ^*JA101*^ *FRT19/FM7*, *ey3*.*5-FLP*, *tubPGAL80*, *FRT19; act-GAL4*,*UAS-lacZ/Cyo*.

### Immunohistochemistry

Fly brains were dissected in PBS and fixed for 1 h in PLP (2% paraformaldehyde, 75 mM Lysine, 37 mM Sodium phosphate buffer, pH 7.4). Eye imaginal discs and pupal retinas were dissected in PBS and fixed in PLP for 20 min. For synapses stained with antibodies against Bruchpilot, fly brains were processed as in [41]. Antibodies and dilutions used were as follows: rabbit anti-βGal (1:5,000 Cappel) (Biogenesis); rat anti-Elav (1:40) (from Developmental Studies Hybridoma Bank [DSHB], clone Rat-Elav-7E8A10); mouse anti-prospero (DSHB clone mouse Pros-MR1A); mouse anti-Bruchpilot (1:50, nc82, DSHB), guinea pig anti-senseless (1:1000, H.J. Bellen), mouse mAb24B10 (1:1000; DSHB), mouse anti-flamingo (1:20; DSHB), rabbit anti-Myc (1:250; Santa Cruz Biotechnology), rabbit anti-capricious (1:2000, A. Nose) and rat anti-otd (1:500, T. Cook). Secondary antibodies were obtained from Jackson ImmunoResearch Laboratories: goat anti-mouse, anti-rabbit, anti-rat, and anti-guinea pig F(ab)2 fragments coupled to FITC, Cy3, or Cy5 (1:200 for FITC and Cy5, and 1:400 for Cy3). Brains, pupal and adult retinas were mounted in Vectashield (Vector Laboratories).

### Quantification of the mistargeting R8 axon terminals

For each brain analysed, the total number of labelled R8 axon terminals that reached the M3 and M6 layers was counted and the percentage of R8 axons mistargeting to M6, the layer in which wild-type R7 axons terminate, was calculated. At least 14 brains for each genotype were analysed. The mean and standard error of the percentage of mutant R8 neurons mistargeting to M6 was calculated. R8 axon terminals that stalled at the more superficial layers were not included in the quantification.

### RT-PCR

RNA extraction on approximately 20 Canton S and 20 *otd* ^*uvi*^ staged retinae was carried out in triplicates for each genotype (40 h APF) were used for each RT-PCR. Real-time PCR was performed using SYBR MESA BLUE Kit (Eurogentec). Oligonucleotides used are listed in S1 Table. Statistical significance was calculated using an unpaired *t-test* with significance at *P*<*0*.*05*.

### Electron microscopy

Electron microscopy was performed as in [42] with a Tecnai G2 Spirit transmission electron microscope (FEI).

## Supporting Information

S1 Fig Developmental stages of R7 and R8 synaptic-layer selection. Confocal microscope images showing the developmental stages of the *Drosophila* visual system stained with the 24B10 antibody that detects photoreceptors and their axons. The total series represents a period of approximately 3 days and progresses (left to right) from the third instar larval stage to adult flies. (**A**) At the third instar larval stage, the outer photoreceptor axons in the eye disc project to form the lamina plexus (L), while the R8 axons project to form the medulla (M). (**B**) At 40 h after puparium formation (APF) the outer photoreceptor axons have projected further into the brain, with the R7 and R8 axons pausing at their temporary layers. (**C**) By ~55 h APF the R7 and R8 axons regain their motility and begin projecting towards their final medulla layers. (**D**) At the adult stage the R7 and R8 photoreceptor axons terminate at their final M6 and M3 medulla layers respectively.
(TIFF)

S2 Fig Fmi expression and Neural Superposition. F-actin is labeled with Phalloidin-TxRed (PHAL, red) and Fmi (green) in the lamina plexus of: wild-type (**A**,**A**”), wild-type animals where *fmi* is overexpressed (**B**,**B”**). (**C**) Electron micrographs showing the lamina of wild-type animals where *fmi* has been overexpressed using the GMR-Gal4 driver. Photoreceptor terminals are colored in pink and numbered. Disrupted cartridges in which the number of afferent axons could not be reliably scored are encircled by a black line. (**D**) Frequency distribution for the number of terminals per cartridge in *GMR-Gal4*; *UAS-fmi* lamina. Of 61 assessed cartridges, 30 (49%) presented a very strong phenotype and could not be scored for their number of afferent axons. Out of 31 cartridges that could be scored, 13 (42%) contained the wild-type complement of 6 axons, which was also the most frequently represent number. The remaining cartridges contain 4 (n = 4, 13%), 5 (n = 7, 23%), 7 (n = 6, 19%) or 8 (n = 1, 3%) axons. The variance in the number of axons is 1.05, which is lower than the one we calculated in *otd* ^*uvi*^ mutant (2.83), *otd* ^*uvi*^ *; GMR-Gal4; UAS-fmi* (1.66) and *otd* ^*uvi*^ *; GMR-Gal4; UAS-gogo* animals (1.70). (**E**,**E”**) *otd* ^uvi^ mutant lamina stained for Phalloidin-TxRed (PHAL, red) and Fmi (green) and *otd* ^uvi^ mutant where *fmi* in provided back using the GMR-Gal4 driver (**F**,**F”**).
(TIFF)

S3 Fig *otd* acts autonomously to the R8. Clonal analysis of the R8 *otd*-mutant phenotype Mosaic animals created using the *eye3*.*5–FLP*/MARCM in which R7 and R8s *otd* ^*JA101*^ mutant cells express LacZ (Red). In the merged panel, R8 cells are labeled using the Rh6-EGFP (green) reporter gene [45] and the *otd* ^*JA101*^ mutant axons are in red. All R8 and R7 axons are visualized with 24B10 antibody (blue). White arrows point to ectopic R8 projection in the M6 layer. A yellow arrow points to a normal projection in the M3 layer.
(TIFF)

S4 Fig otd is required in R8 to promote axon projection to the M3 layer. (**A**) Real-time PCR quantification of *otd* mRNA in *otd* mutant retina and optic lobes at 40% after puparium formation. Transcript levels were normalized against wild-type and *GAPDH* mRNA levels. *n* = at least three independent mRNA extracts from wild-type and *otd-*mutant retinas and optic lobes. Error bars represent SEM. (**B,C**) Expression of two distinct *UAS-otd* ^*RNAi*^ (lines 29342 and 34327) transgenes (GFP-negative ommatidia, encircled by a dotted line) in wild-type tissue (GFP positive) 48 h after clone induction using the *tub>GFP>Gal4* system. In both RNAi lines, *UAS-otd* ^*RNAi*^ expressing cells show a clear reduction in Otd protein levels (red), while Sens expression (blue) remains unchanged. (**D,E**) Rh6-lacZ-positive R8 axons in two distinct *otd* ^*RNAi*^ lines (GMRGal4;*UAS*-*otd* ^*RNAi*^) stained with anti-β-galactosidase (green). Both *otd* ^*RNAi*^ lines (29342 and 34327) show the same R8 misprojection phenotype seen in *otd* ^*uvi*^ mutants. Arrows point to R8 axons misprojecting to the M6 layer.
(TIFF)

S5 Fig otd-mutant R8 form synapses in the M6 synaptic-layer. (**A**) In *otd* ^*uvi*^ mutant adult optic lobe, Bruchpilot protein (green) co-localizes with ectopic R8 terminals (*Rh6-lacZ*, *red*) in the M6 layer (arrows). Magnified pictures in (**A’**) and (**A”**). In wild-type (**B**) Rh6-lacZ-positive R8 axons (in red) make synapses (visualized by UAS-BRP-GFP driven by R8Gal4) in the M3 layer. (**C**) *otd* ^*uvi*^ mutant R8 axons that misproject to the M6 layer also form synapses in this layer (colocalization between the GFP signal and R8 terminals). Arrows point to misprojecting R8 terminals that synapse in the M6 layer. Photoreceptor cell projections are stained with 24B10 (blue).
(TIFF)

S6 Fig otd promotes the expression of caps, gogo and fmi. Real-time PCR quantification of *caps*, *gogo* and *fmi* mRNA in *otd* ^*uvi*^ mutant retina (40% after puparium formation) in which *otd* has been re-introduced using the GMRGal4 driver. GAPDH was used as the reference gene and transcript levels normalized to wild-type levels. The pupae were selected at 40% after puparium formation. n = three independent mRNA extracts. Error bars represent SEM. Re-introducting *otd* expression in *otd* ^uvi^ mutant retina cells restores the expression of these CAMs to wild-type levels or higher (with fold-changes relative to wild-type of 1.16 ± 0.11 [p>0.05], 1.80 ± 0.10 [p<0.05] and 1.67 ± 0.13 [p<0.05] for *caps*, *gogo* and *fmi* respectively).
(TIFF)

S7 Fig Overexpression of *otd* does not affect R7 and R8 synaptic-layer projection. Adult optic lobes from GMR*-otd flies* expressing the R8 specific marker Rh6-lacZ (A) and the R7 specific marker Rh3-lacZ (B). R8 and R7 terminate normally in the M3 and M6 layer respectively. Photoreceptor cell projections are stained with 24B10 antibody (red).
(TIFF)

S1 Table List of oligonucleotides used to perform RT-PCR experiments. (TIF)
