# Supplementary figures and images for: Orthodenticle Is Required for the Expression of Principal Recognition Molecules That Control Axon Targeting in the Drosophila Retina
Source: PLoS Genet. 2015 Jun 26;11(6):e1005303. doi: 10.1371/journal.pgen.1005303 (PMC4482733; doi:10.1371/journal.pgen.1005303)

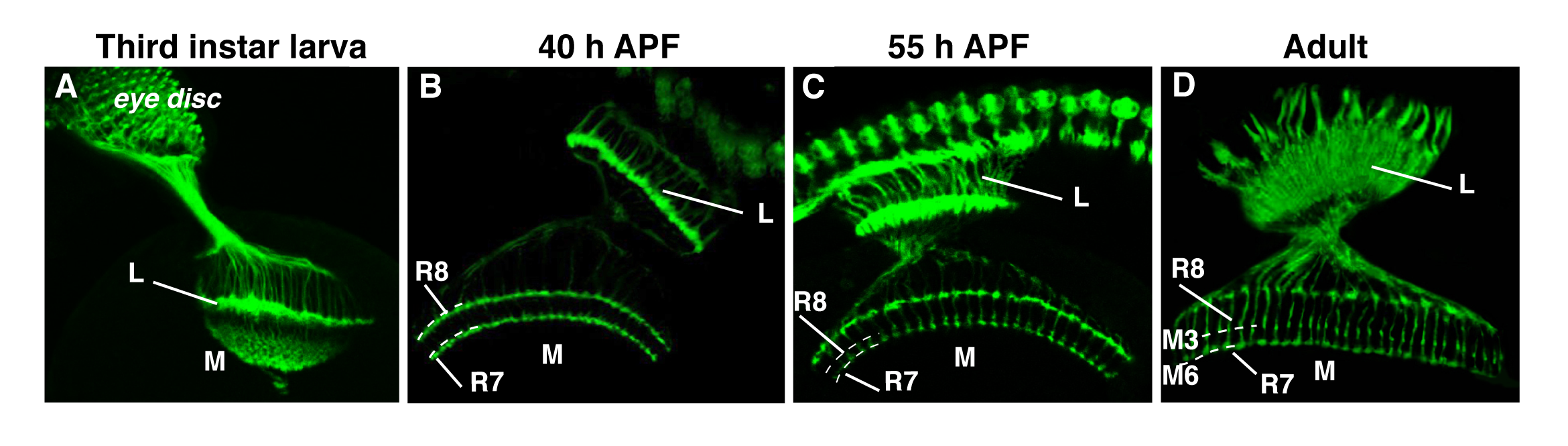

Supplement: S1 Fig — Confocal microscope images showing the developmental stages of the Drosophila visual system stained with the 24B10 antibody that detects photoreceptors and their axons. The total series represents a period of approximately 3 days and progresses (left to right) from the third instar larval stage to adult flies. (A) At the third instar larval stage, the outer photoreceptor axons in the eye disc project to form the lamina plexus (L), while the R8 axons project to form the medulla (M). (B) At 40 h after puparium formation (APF) the outer photoreceptor axons have projected further into the brain, with the R7 and R8 axons pausing at their temporary layers. (C) By ~55 h APF the R7 and R8 axons regain their motility and begin projecting towards their final medulla layers. (D) At the adult stage the R7 and R8 photoreceptor axons terminate at their final M6 and M3 medulla layers respectively. (TIFF) [file pgen.1005303.s001.tiff]

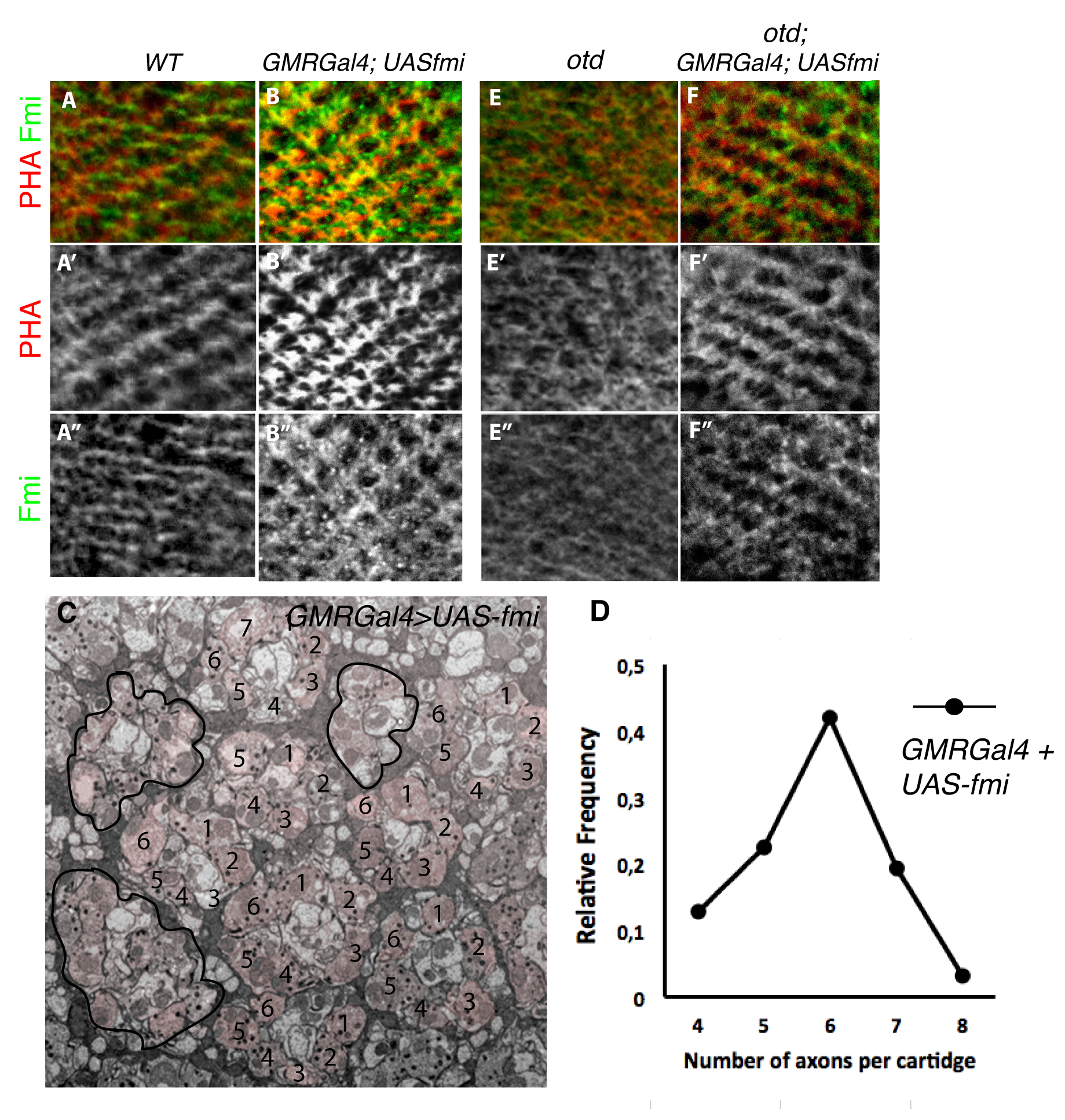

Supplement: S2 Fig — F-actin is labeled with Phalloidin-TxRed (PHAL, red) and Fmi (green) in the lamina plexus of: wild-type (A,A”), wild-type animals where fmi is overexpressed (B,B”). (C) Electron micrographs showing the lamina of wild-type animals where fmi has been overexpressed using the GMR-Gal4 driver. Photoreceptor terminals are colored in pink and numbered. Disrupted cartridges in which the number of afferent axons could not be reliably scored are encircled by a black line. (D) Frequency distribution for the number of terminals per cartridge in GMR-Gal4; UAS-fmi lamina. Of 61 assessed cartridges, 30 (49%) presented a very strong phenotype and could not be scored for their number of afferent axons. Out of 31 cartridges that could be scored, 13 (42%) contained the wild-type complement of 6 axons, which was also the most frequently represent number. The remaining cartridges contain 4 (n = 4, 13%), 5 (n = 7, 23%), 7 (n = 6, 19%) or 8 (n = 1, 3%) axons. The variance in the number of axons is 1.05, which is lower than the one we calculated in otd uvi mutant (2.83), otd uvi ; GMR-Gal4; UAS-fmi (1.66) and otd uvi ; GMR-Gal4; UAS-gogo animals (1.70). (E,E”) otd uvi mutant lamina stained for Phalloidin-TxRed (PHAL, red) and Fmi (green) and otd uvi mutant where fmi in provided back using the GMR-Gal4 driver (F,F”). (TIFF) [file pgen.1005303.s002.tiff]

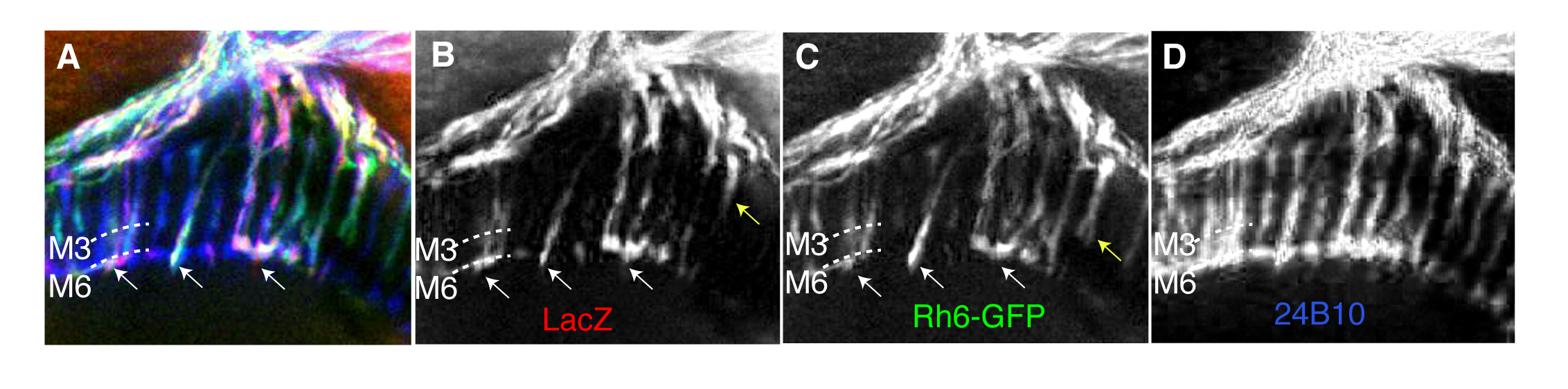

Supplement: S3 Fig — Clonal analysis of the R8 otd-mutant phenotype Mosaic animals created using the eye3.5–FLP/MARCM in which R7 and R8s otd JA101 mutant cells express LacZ (Red). In the merged panel, R8 cells are labeled using the Rh6-EGFP (green) reporter gene [45] and the otd JA101 mutant axons are in red. All R8 and R7 axons are visualized with 24B10 antibody (blue). White arrows point to ectopic R8 projection in the M6 layer. A yellow arrow points to a normal projection in the M3 layer. (TIFF) [file pgen.1005303.s003.tiff]

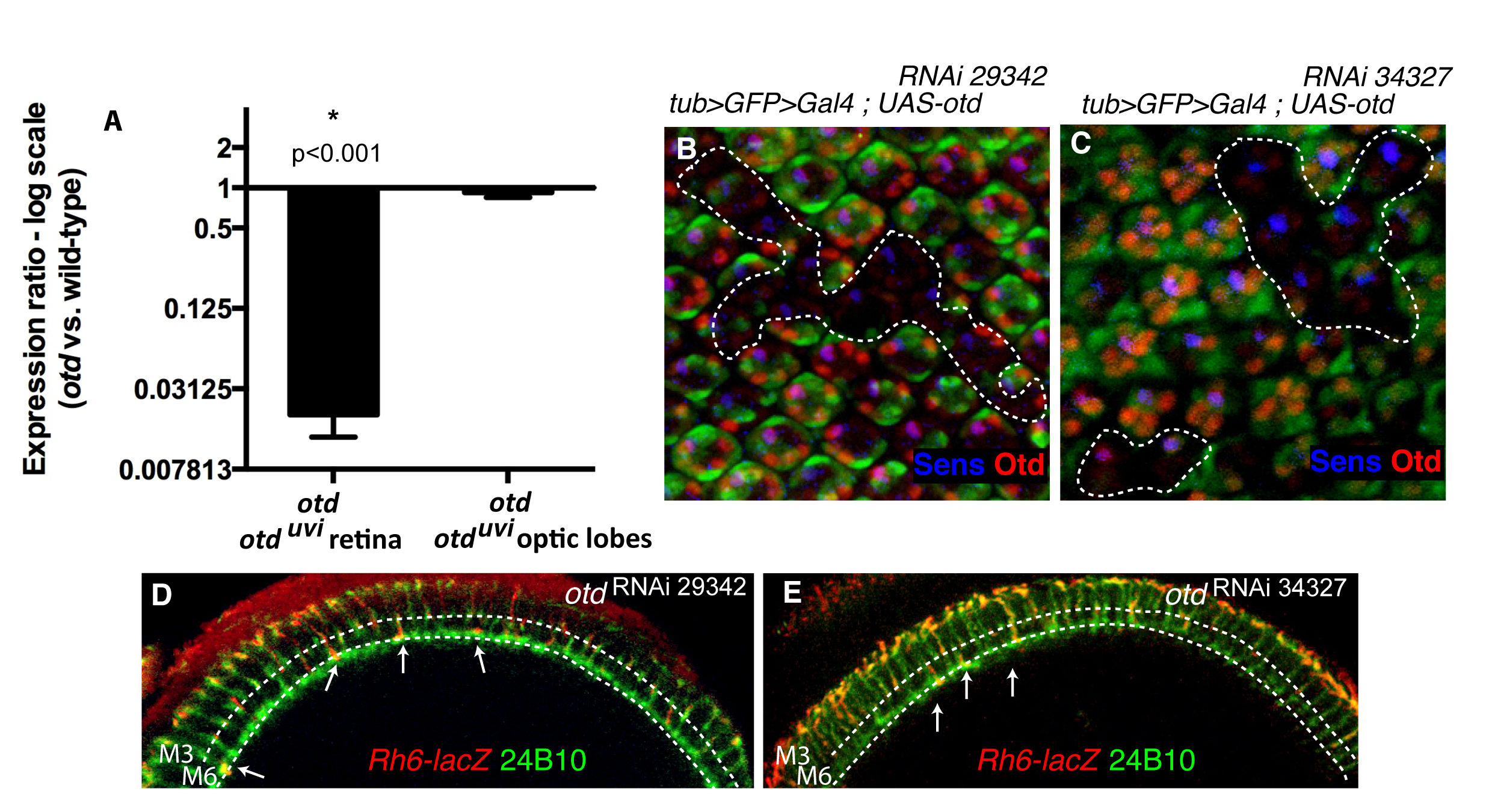

Supplement: S4 Fig — (A) Real-time PCR quantification of otd mRNA in otd mutant retina and optic lobes at 40% after puparium formation. Transcript levels were normalized against wild-type and GAPDH mRNA levels. n = at least three independent mRNA extracts from wild-type and otd-mutant retinas and optic lobes. Error bars represent SEM. (B,C) Expression of two distinct UAS-otd RNAi (lines 29342 and 34327) transgenes (GFP-negative ommatidia, encircled by a dotted line) in wild-type tissue (GFP positive) 48 h after clone induction using the tub>GFP>Gal4 system. In both RNAi lines, UAS-otd RNAi expressing cells show a clear reduction in Otd protein levels (red), while Sens expression (blue) remains unchanged. (D,E) Rh6-lacZ-positive R8 axons in two distinct otd RNAi lines (GMRGal4;UAS-otd RNAi) stained with anti-β-galactosidase (green). Both otd RNAi lines (29342 and 34327) show the same R8 misprojection phenotype seen in otd uvi mutants. Arrows point to R8 axons misprojecting to the M6 layer. (TIFF) [file pgen.1005303.s004.tiff]

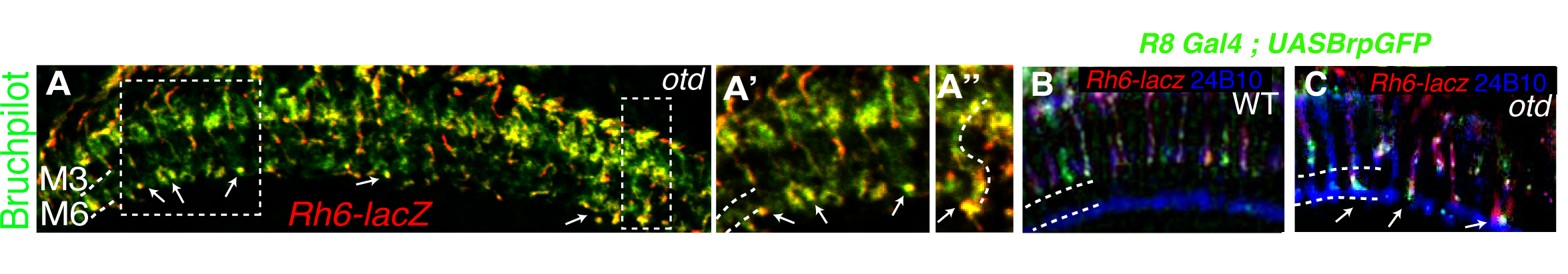

Supplement: S5 Fig — (A) In otd uvi mutant adult optic lobe, Bruchpilot protein (green) co-localizes with ectopic R8 terminals (Rh6-lacZ, red) in the M6 layer (arrows). Magnified pictures in (A’) and (A”). In wild-type (B) Rh6-lacZ-positive R8 axons (in red) make synapses (visualized by UAS-BRP-GFP driven by R8Gal4) in the M3 layer. (C) otd uvi mutant R8 axons that misproject to the M6 layer also form synapses in this layer (colocalization between the GFP signal and R8 terminals). Arrows point to misprojecting R8 terminals that synapse in the M6 layer. Photoreceptor cell projections are stained with 24B10 (blue). (TIFF) [file pgen.1005303.s005.tiff]

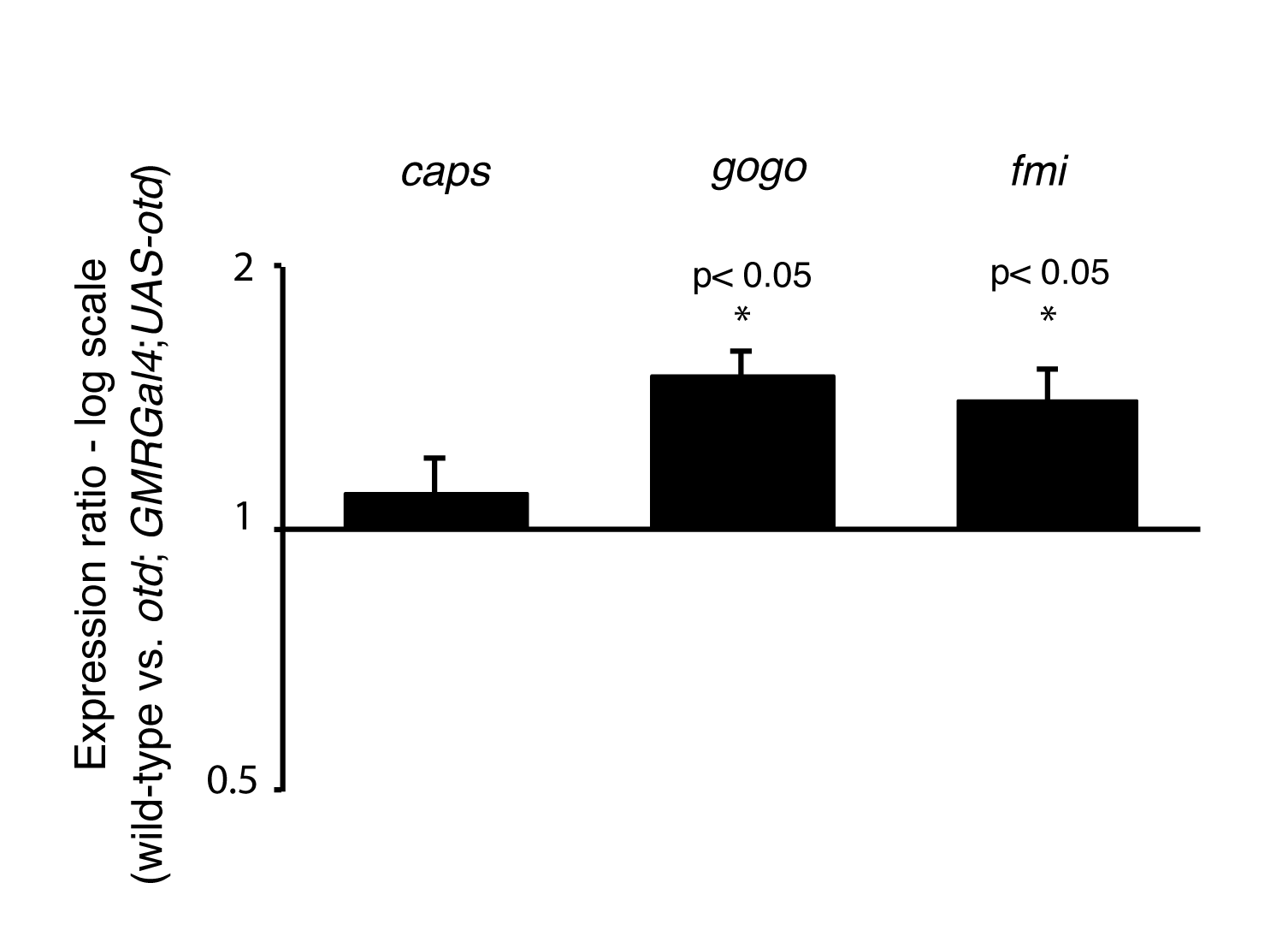

Supplement: S6 Fig — Real-time PCR quantification of caps, gogo and fmi mRNA in otd uvi mutant retina (40% after puparium formation) in which otd has been re-introduced using the GMRGal4 driver. GAPDH was used as the reference gene and transcript levels normalized to wild-type levels. The pupae were selected at 40% after puparium formation. n = three independent mRNA extracts. Error bars represent SEM. Re-introducting otd expression in otd uvi mutant retina cells restores the expression of these CAMs to wild-type levels or higher (with fold-changes relative to wild-type of 1.16 ± 0.11 [p>0.05], 1.80 ± 0.10 [p<0.05] and 1.67 ± 0.13 [p<0.05] for caps, gogo and fmi respectively). (TIFF) [file pgen.1005303.s006.tiff]

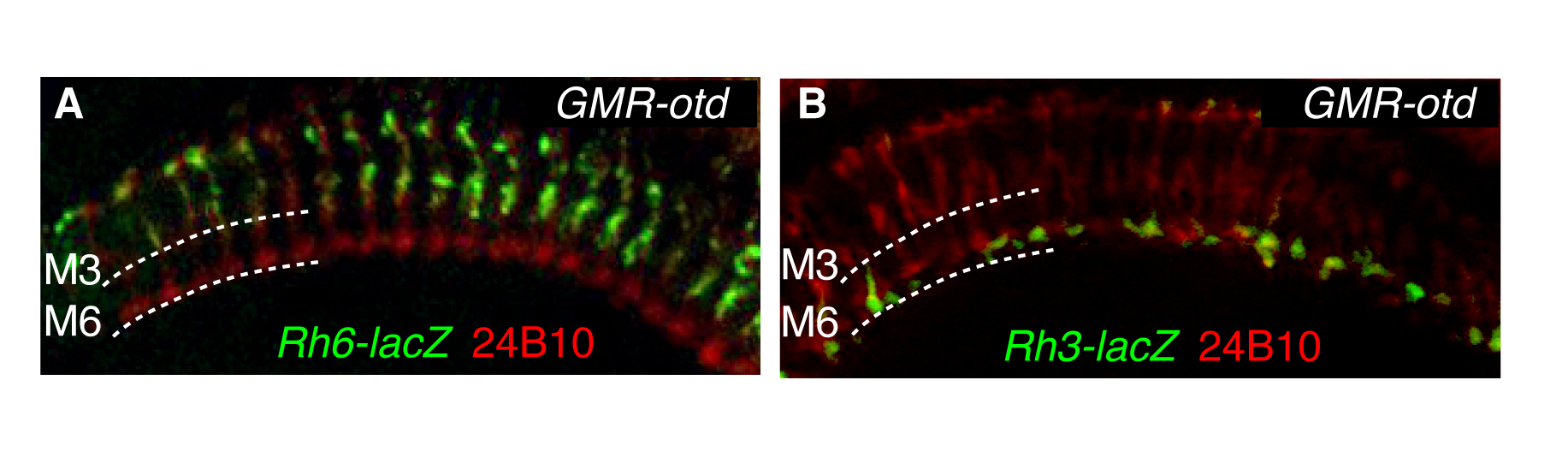

Supplement: S7 Fig — Adult optic lobes from GMR-otd flies expressing the R8 specific marker Rh6-lacZ (A) and the R7 specific marker Rh3-lacZ (B). R8 and R7 terminate normally in the M3 and M6 layer respectively. Photoreceptor cell projections are stained with 24B10 antibody (red). (TIFF) [file pgen.1005303.s007.tiff]

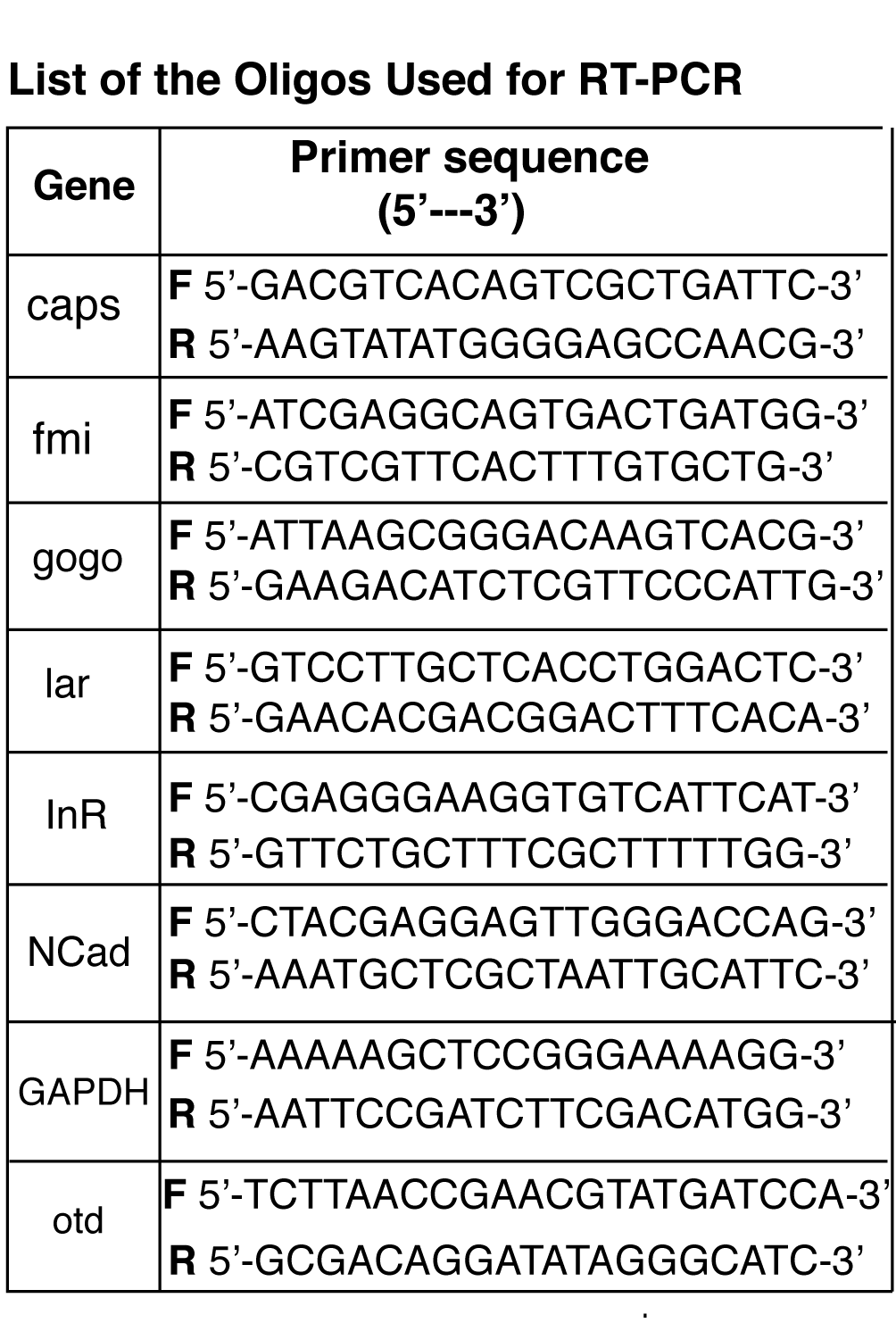

Supplement: S1 Table — (TIF) [file pgen.1005303.s008.tif]
